# Penetrable Nanoplatform for “Cold” Tumor Immune Microenvironment Reeducation

**DOI:** 10.1002/advs.202000411

**Published:** 2020-07-29

**Authors:** Qinjun Chen, Yongqing He, Yu Wang, Chao Li, Yujie Zhang, Qin Guo, Yiwen Zhang, Yongchao Chu, Peixin Liu, Hongyi Chen, Zheng Zhou, Wenxi Zhou, Zhenhao Zhao, Xiaomin Li, Tao Sun, Chen Jiang

**Affiliations:** ^1^ Key Laboratory of Smart Drug Delivery (Ministry of Education) State Key Laboratory of Medical Neurobiology and MOE Frontiers Center for Brain Science Institutes of Brain Science Department of Pharmaceutics School of Pharmacy Research Center on Aging and Medicine Fudan University Shanghai 201203 P. R. China; ^2^ Department of Chemistry and Laboratory of Advanced Materials Fudan University Shanghai 200433 P. R. China

**Keywords:** antitumor immune responses, immunogenic cell death, immunosuppression, metal–organic frameworks, penetration

## Abstract

Lack of tumor‐infiltration lymphocytes (TILs) and resistances by overexpressed immunosuppressive cells (principally, myeloid‐derived suppressor cells (MDSCs)) in tumor milieu are two major challenges hindering the effectiveness of immunotherapy for “immune‐cold” tumors. In addition, the natural physical barrier existing in solid cancer also limits deeper delivery of drugs. Here, a tumor‐targeting and light‐responsive‐penetrable nanoplatform (Apt/PDGs_^_s@pMOF) is developed to elicit intratumoral infiltration of cytotoxic T cells (CTLs) and reeducate immunosuppressive microenvironment simultaneously. In particular, porphyrinic metal–organic framework (pMOF)–based photodynamic therapy (PDT) induces tumor immunogenic cell death (ICD) to promote CTLs intratumoral infiltration and hot “immune‐cold” tumor. Upon being triggered by PDT, the nearly 10 nm adsorbed drug‐loaded dendrimer de‐shields from the nanoplatform and spreads into the deeper tumor, eliminating MDSCs and reversing immunosuppression, eventually reinforcing immune response. Meanwhile, the designed nanoplatform also has a systemic MDSC inhibition effect and moderate improvement of overall antitumor immune responses, resulting in effective suppression of distal tumors within less significant immune‐related adverse effects (irAEs) induced.

## Graphical abstract

A penetrable nanoplatform is developed to elicit intratumoral infiltration of cytotoxic T cells (CTLs) and reeducate immunosuppressive microenvironment simultaneously. In particular, immunogenic cell death induced by photodynamic therapy (PDT) promotes CTLs infiltration, upon being triggered by PDT, drug‐loaded dendrimer spreads into deeper tumor, eliminating myeloid‐derived suppressor cells and reversing immunosuppression, eventually reinforcing immune responses.

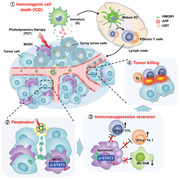

## 1 Introduction

Triple‐negative breast cancer (TNBC) is a highly aggressive subtype of breast cancers with poor prognosis and no approved targeted therapy available other than conventional chemotherapy.^[^ ^1^ ^]^ Immunotherapy, an established new pillar of treatments for other cancers, has shown an impressive treatment outcome in partial TNBC patients.^[^ ^2^ ^]^ However, nearly 80% of TNBC patients are diagnosed with low or no infiltration of cytotoxic T cells (CTLs) in tumor lesions, also called “cold” tumor, making the checkpoint inhibitors‐based therapies invalid.^[^ ^3^ ^]^ Cancer vaccine has been regarded as an effective agent for systemic antitumor immune enhancement and tumor‐infiltration of CTL in the past decades.^[^ ^4^ ^]^ However, mentioned in recent reviews, the immune‐related adverse effects (irAEs) and resistances by existing immunosuppressive cells (likes myeloid‐derived suppressor cells (MDSCs)) in tumor milieu made most cancer vaccines failing to accomplish an objective antitumor activity.^[^ ^5^ ^]^ Thus, therapeutic approach to elicit intratumoral infiltration of CTLs and simultaneously reverse the resistances by immunosuppressive cells, as well as reduced irAEs, may be beneficial for effective anti‐TNBC immunotherapy.

Immunogenic cell death (ICD) is a special type of cell death that can convert residual cellular pieces into a regional intensive vaccine to reinforce antitumor CTL infiltration.^[^ ^6^ ^]^ Particularly, surface‐translocated calreticulin (CRT) serves as an “eat me” signal for dendritic cell (DC) phagocytosis, milieu‐released high mobility group protein B1 (HMGB1) promotes DC maturation and antigen‐presentation to CTLs, as well as secreted ATP stimulates the intratumoral CTLs infiltration.^[^ ^7^ ^]^ It is now accepted that the micro‐invasive photodynamic therapy (PDT) had exhibited a superior capacity of reactive oxygen species (ROS)‐related ICD induction.^[^ ^8^ ^]^ However, most of frequently used photosensitizers (such as phthalocyanines and porphyrins) are hydrophobic and tend to be aggregated in aqueous solution, resulting in reduced photodynamic yield and insufficient efficacy toward tumor lesions.^[^ ^9^ ^]^ Porphyrinic metal–organic framework (pMOF), the latest generation of PDT agents, has been demonstrated that could keep the photosensitizer in monomeric form and prevent its self‐quenching.^[^ ^10^ ^]^ In addition, the nanoscaled crystal size and modifiable surface functional groups of pMOF can favor porphyrin tumor accumulation and PDT synergy.^[^ ^11^ ^]^ Therefore, ICD induced by pMOF‐based PDT would be a hopeful way for increasing CTLs infiltration in TNBC lesions.

MDSCs are the major host component participating in the immunosuppressive tumor microenvironment (TIME) and disrupt the host immune‐surveillance through various restraining mechanisms, such as direct T cell inhibition, M1 macrophage impairment, and programmed death ligand‐1 (PD‐L1) upregulation.^[^ ^12^ ^]^ Compared with healthy controls, MDSCs are abundant in TNBC patients, especially having an increased tumor MDSC infiltration and promoting tumor progression.^[^ ^13^ ^]^ Activation of Janus kinase/signal transducers and activators of transcription 3 (JAK/STAT3) pathway plays a responsible role in the formation and expansion of MDSCs, and several in‐depth studies reported that gemcitabine (GEM) could preferentially reduce the percentages of MDSCs without other leukocytes eliminated via the selective blockade of JAK/STAT3 pathway in MDSCs.^[^ ^14^ ^]^ However, due to the short blood half‐life and lack tumor accumulation of GEM, its intratumoral therapeutic action was restricted.^[^ ^15^ ^]^ To solve these problems, various nano drug delivery systems, always combined with prodrug strategies, were widely developed and some of them have shown an enhanced antitumor efficacy.^[^ ^16^ ^]^ In addition, of note, there is usually a natural physical barrier existing in solid cancer that blocks the deeper penetration of nanoparticles, while just nanoparticles with a particle size less than 30 nm may can accomplish a better penetration.^[^ ^17^ ^]^ In this study, we loaded GEM‐prodrug to a nearly 10 nm size cationic dendrimer (PEG‐DGL) and expected the designed DGL could carry GEM‐prodrug into the deeper tumor region to deplete the tumor‐infiltrated MDSCs effectively.

Herein, we report on a layer‐by‐layer Apt/PDGs_^_s@pMOF nanoparticle loading with GEM‐prodrug for increasing the intratumoral CTL infiltration and TIME reeducation synergistically. To improve the tumor‐targeting ability of designed nanoparticle, a stroma and tumor dual‐targeting ligand, periostin‐targeting DNA aptamer (Apt), was introduced for active‐targeting.^[^ ^18^ ^]^

## 2 Results and Discussion

### 2.1 Preparation and Characterization of Apt/PDGs_^_s@pMOF

**Scheme 1:**
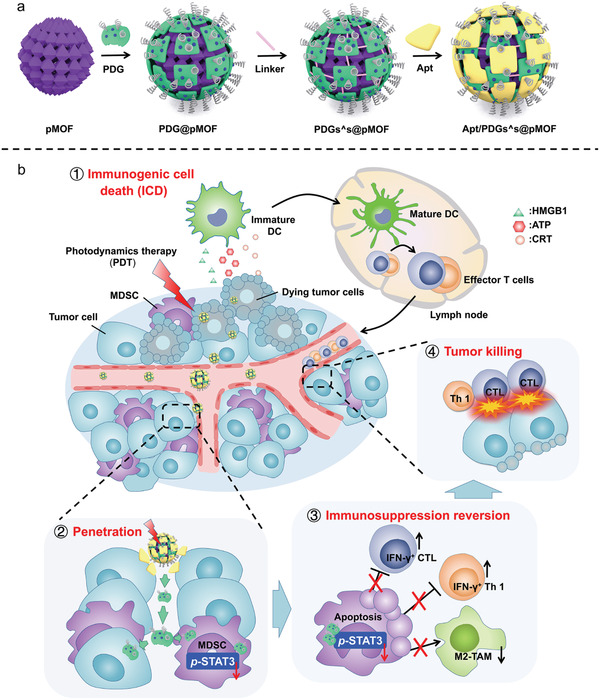
Representation of a) preparation and b) elevated antitumor immune response with penetrable nanoplatform treatment.

As designed, the formulation was composed of three parts, pMOF core, GEM‐loaded DGLs shells (PDG) and periostin‐targeting aptamer layers (Apt) (**Scheme** **1**a). In detail, via electrostatic attraction, the electropositive GEM‐loaded DGL was adsorbed to the negative surface of pMOF to form PDG@pMOF. Next, an ROS‐sensitive crosslinking was introduced to strengthen the electrostatic interaction, named PDGs_^_s@pMOF. In the end, periostin‐targeting DNA aptamer (Apt) was coated onto PDGs_^_s@pMOF, and final layer‐by‐layer formulation, Apt/PDGs_^_s@pMOF, was obtained. We first explored the synthesis of meso‐tetra (4‐carboxyphenyl) porphine, PDG shell and ROS‐sensitive crosslinker (Scheme S1, Supporting Information). Through a variety of chemical conjugation including ring‐opening reaction, amidation reaction, and click reaction, we successfully obtained the PDG shell and other key compounds, which were characterized by proton nuclear magnetic resonance spectra (^1^H NMR), mass spectrometry (MS), gel permeation chromatography (GPC), illustrated in Figures S1 and S12 in the Supporting Information. The freshly prepared pMOF nanoparticles were found with uniform size in water by dynamic light scattering (DLS) and transmission electron microscopy (TEM).

**Figure 1:**
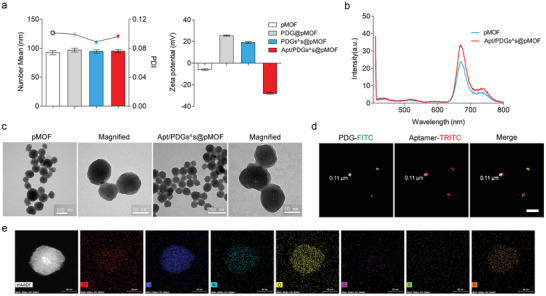
Characterization of Apt/PDGs^s@pMOF formulations. a) Variation of DLS profile and Zeta potential in the lay‐by‐lay process of Apt/PDGs^s@pMOF preparation. b) Fluorescence emission spectrum excited at 405 nm of pMOF before and after capped with PDG and Aptamer. c) TEM images of pMOF and Apt/PDGs^s@pMOF. d) Co‐localization of PDG‐FITC and Aptamer‐TRITC under Leica TCS SP8 STED. ex/em _(PDG‐FITC)_: 488 nm/520 nm. ex/em _(Aptamer‐TRITC)_: 557 nm/576 nm. Scale bars: 1 µm. e) HAADF image and the distribution for the elemental mapping of Zr, C, N, O, S, F, P. Scale bars: 30 nm.

Next, the preparation process of Apt/PDGs_^_s@pMOF was investigated meticulously. As shown in **Figure** **1**a and Figures S13 and S14 in the Supporting Information, the averaged hydrodynamic diameter of nanoparticle and polydispersity index (PDI) value were maintained at around 100 nm and 0.1, respectively, showing a fine water‐dispersion. At the same time, the zeta potential of nanoparticle in preparation was followed with the electroconductivity variation of the adsorbed materials, firstly was −6.47 mV, then reversely shifted to +24.9 mV substantially within PDG adhesion, then slightly decreased to +20.0 mV after crosslinking, and finally converted to −27.5 mV after envelopment with Apt. In addition, Apt/PDGs_^_s@pMOF had maintained fine particle diameters during incubation with dulbecco's modified eagle medium (DMEM, containing 10% foetal bovine serum (FBS)) for 7 day (Figure S13d–f, Supporting Information). This experimental phenomenon indicated the successful layer‐by‐layer processes and the moderately negative zeta potential of final formulation would lead to high colloidal stability on water.^[^ ^19^ ^]^ As shown in Figure 1b, the fluorescence spectrum of final preparation and pMOF were almost consistent, revealing that the layer‐by‐layer packaging process did not affect the property of pMOF. TEM results also showed that pMOF was spherical in shape with an average morphological diameter of 70 nm (Figure 1c). Compared with pMOF, there was a clear and thick organic layer at the outermost edge of Apt/PDGs_^_s@pMOF (owing to the fact that the principal constituents of pMOF was organic materials, a thin organic layer could be also observed in TEM photograph of pMOF), as well as the clear lattice gap in pMOF was blurred due to the PDG wrapping. To further demonstrate that the formation of Apt/PDGs_^_s@pMOF was induced by layer‐by‐layer, we labeled the PDG with fluorescein isothiocyanate (FITC) and aptamer with tetraethyl rhodamine isothiocyanate (TRITC), and took fluorescence photographs of nanoparticles under stimulated emission depletion microscopy (STEM). As illustrated in Figure 1d, the green fluorescence of FITC displayed a moderate co‐localization with the red fluorescence of TRITC (because of the unavoidable Brownian motion, it was impossible to achieve a complete co‐localization), with a particle size at around 110 nm, which was close to the DLS result. These results intuitively testified that aptamer was tightly bound to PDG. Fortunately, we further demonstrated that aptamer was strongly combined with pMOF via the elemental mapping technique of STEM (Figure 1e), showing a centralized mapping‐co‐localization of P elemental (representing aptamer) and Zr elemental (representing pMOF). Moreover, the STEM mapping results of drug‐free Apt/PDs_^_s@pMOF showed that the mapping of S elemental (represented crosslinker) was co‐localized with that of Zr elemental (represented pMOF) while the interference of sulfhydryl groups was excluded (Figure S13g, Supporting Information), proving the existence of crosslinking. At this point, the layer‐by‐layer Apt/PDGs_^_s@pMOF was completely accomplished as designed in advance. Meanwhile, these results also suggested that the designed delivery system was not restricted by its loading‐drug, kinds of drugs could be introduced into this system. Finally, low hemolysis was also observed for the Apt/PDGs^s@pMOF nanoparticle (Figure S15, Supporting Information).

### 2.2 Investigation on PDT Properties and Cellular Uptake of Nanoparticles

**Figure 2:**
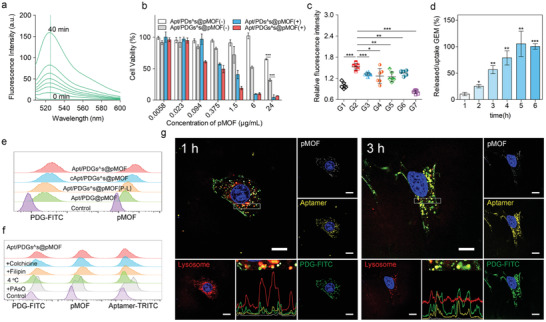
Investigation on PDT properties and cellular uptake of Apt/PDGs^s@pMOF. a) Florescence response of H_2_DCFH‐DA upon treatment with pMOF. Irradiation time from bottom to up was from 0 to 40 min. ex 485 nm. b) CCK‐8 assay of 4T1 cells after 48 h treatment with various concentrations of different formulations within (+) or without (−) irradiation. Data are presented as means ± SD (*n* = 4). c) Hydroxyl radical generation in 4T1 cells upon PDT treatment with different formulations. Data are presented as means ± SD (*n* = 6). d) Time‐dependent variation of intracellular PDG‐FITC uptake ratio and released/uptake ratio of GEM. Data are presented as means ± SD (*n* = 3). e) Cellular uptake of different formulations on 4T1 cells via flow cytometry analysis, based on the fluorescence signal of FITC‐labeled PDG and pMOF, respectively. f) Flow cytometry analysis of Apt/PDGs^s@pMOF's internalization mechanism, based on the fluorescence signal of FITC‐labeled PDG, TRITC‐labeled Aptamer and pMOF. g) Co‐localization of Apt/PDGs^s@pMOF and lysosome in 4T1 cells under CLSM after 1 h treatment, and a decreased co‐localization was found after 3 h treatment. Scale bars: 15 µm. G1: Control +L (laser irradiation), G2: Apt/PDGs^s@pMOF + L, G3: cApt/PDGs^s@pMOF + L, G4: Apt/PDG@pMOF + L, G5: Apt/PDGs^s@pMOF (P‐L, pre‐irradiated) + L, G6: Apt/PDGs^s@pMOF (+NAC) + L, G7: Apt/PDGs^s@pMOF + D (dark). Significance is defined as ns, no significance, ^*^ *p* < 0.05, ^**^ *p* < 0.01, ^***^ *p* < 0.001.

After the successful preparation of Apt/PDGs_^_s@pMOF, we used 2′, 7′‐dichlorofluorescein diacetate (H_2_DCFH‐DA) as a ROS probe to evaluate the PDT properties of Apt/PDGs_^_s@pMOF.^[^ ^20^ ^]^ As shown in **Figure** **2**a, the irradiation time‐dependent increase in fluorescence emission at 525 nm indicated a favorable photo‐induced ROS generation by Apt/PDGs_^_s@pMOF. Then, we evaluated the cytotoxicity of the nanoparticles with or without GEM‐loading. The IC_50_ of Apt/PDs_^_s@pMOF was at nearly 25.61 µg mL^−1^ when shielded from light, and with a threefold decline upon GEM introduction. Meanwhile, when irradiated with 660 nm light‐emitting diode (LED) light (30 mW cm^−2^) for 5 min, the IC_50_ value of nanoparticles showed a drop of more than 20 times, which adequately reflected the efficiency of PDT treatment (Figure 2b and Figure S16c, Supporting Information). Subsequently, investigation of ROS generation in cells was carried out (Figure 2c). Upon irradiation, all formulations showed a significant increase in intracellular ROS levels than control (G1) or light‐shielded (G7) group, among which dual tumor‐targeting and crosslinked group (G2) exhibited the highest level of ROS induction. And the moderate ROS levels found in untargeted group (G3), un‐crosslinked group (G4) and light‐pretreated group (G5) would be associated with the insufficient cell uptake detected in those groups than G2 group. Moreover, a ROS inhibitor, *N*‐acetyl‐l‐cysteine (NAC), was used as a negative control (G7), and flow analysis results also supported our findings.

In addition, we testified that the pro‐drug formulation had a sustained drug release capacity in phosphate buffer saline (PBS) 7.4, with only nearly 50% of loaded‐drug release at 96 h (Figures S16e and S17, Supporting Information). Then, an intracellular drug release study was performed and the increased uptake/release ratio of GEM in 4T1 cells during next 6 hours revealed a rapid intracellular drug release (Figure 2d and Figure S16f, Supporting Information). This phenomenon might be due to the hydrolysis by intracellular enzyme which had been reported in several literatures.^[^ ^16^ ^]^

Subsequently, addition to the weak fluorescence emission of pMOF at 660 nm (ex 405 nm), we further labeled PDG polymer with FITC to investigate the overall uptake of Apt/PDGs_^_s@pMOF in 4T1 cells. The ratio of FITC to pMOF fluorescence could be considered as an evaluation index for overall uptake of nanoparticles. Results showed that aptamer covering could notably decrease the whole nanoparticle uptake by Raw264.7 (Figure S18, Supporting Information), and dual periostin‐targeting modification and ROS‐sensitive crosslinking were effective for uptake enhancement in 4T1 cells (Figure 2e and Table S1, Supporting Information). As expected, the FITC/pMOF ratio between Apt/PDGs_^_s@pMOF and non‐targeted cApt/PDGs_^_s@pMOF was slightly shifted from 0.62 to 0.70, suggesting the lessened‐targeting associated uptake decrease in non‐targeted group was performed as a whole. In contrast, whether the FITC/pMOF ratio of pre‐irradiated Apt/PDGs_^_s@pMOF (P‐L) or un‐crosslinked Apt/PDG@pMOF was markedly increased to 0.82 or 1.03, respectively, indicating that the integrity of nanoparticle would be decreased while the crosslinking was cleaved to lead a reduced integral uptake. Besides, similar results were also obtained under confocal laser scanning microscopy (CLSM) observation (Figure S19, Supporting Information).

To investigate the endocytosis mechanism of Apt/PDGs_^_s@pMOF, aptamer was labeled with TRITC, PDG was labeled with FITC, and 4T1 cells were pretreated with several endocytic inhibitors (Figure 2f). According to the flow cytometry data acquired from FITC and pMOF channels, 4 °C and Phenylarsine oxide(PAsO) showed the most significant uptake inhibition, revealing that the endocytosis of Apt/PDGs_^_s@pMOF was energy‐dependent and through the clathrin‐mediated pathway, a classic receptor‐mediated endocytosis pathway.^[^ ^21^ ^]^ As expected, the TRITC/pMOF ratio was somehow increased nearly fourfold in PAsO‐pretreated group while the FITC/pMOF ratio of all groups was on small‐scale fluctuations (Table S2, Supporting Information), suggesting a stable extracellular state of PDGs_^_s@pMOF during experiment, while aptamer layer seemed to be separated from nanoparticle when the uptake was suppressed. It is deduced that the attachment of Apt/PDGs_^_s@pMOF extracellular surface was still under way via aptamer recognition whether endocytosis was inhibited or not. However, the configuration change of aptamer during recognition impair the interaction with PDGs_^_s@pMOF, resulting in de‐shielding of the adsorbed PDGs_^_s@pMOF and leaving aptamer on the cell surface.^[^ ^22^ ^]^ These results also provided an indirect theoretical basis for the secondary tumor‐targeting of designed nanoparticle after having targeted to stromal periostin.

Most formulations that are ingested via receptor‐mediated endocytosis will be normally metabolized by cells through a lysosome pathway. Therefore, lysosomal escape would be a critical capability for expanding the curative of formulations. As shown in Figure 2g, at 1 h, almost all three‐type fluorescence of nanoparticles was collocated with strong lysosome staining, suggesting that the nanoparticle was ingested into the lysosome as a whole and the integrity of lysosome was still well. After incubation for 3 h, the intensity of lysosomal staining was decreased significantly while the nanoparticles’ signals were gradually increased, within the reduced co‐localization to lysosome. Here, we deemed that nanoparticles had accomplished lysosomal escape, which would be due to the classic “proton sponge” effect of PDG in Apt/PDGs^s@pMOF, leading to lysosome bursting and nanoparticle release. In addition, the fluorescence intensity of pMOF was weakened after 3 h incubation, suggesting a pMOF degradation.

### 2.3 Evaluation of ICD Induction and MDSCs Elimination

**Figure 3:**
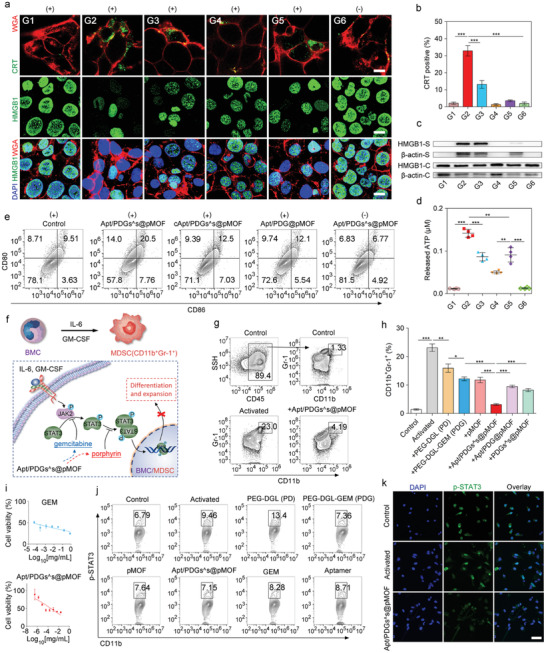
Evaluation of ICD induction and MDSCs elimination. a) CRT exposure and HMGB1 release in 4T1 cells treated with different formulations, following by CLSM. Scale bar: 10 µm. b) Flow cytometry analysis of CRT exposure in different groups. Data are presented as means ± SD (*n* = 3). c) Western blotting analysis of HMGB1 release from 4T1 cells in different groups (S: supernatant, C: cells). d) ATP secretion from different formulation‐treated 4T1 cells. e) Flow cytometry analysis of DCs maturation induced by treated 4T1 cells. f) Illustration of MDSCs inhibition. g) Successful formulation and expansion of MDSCs from IL‐6 and GM‐CSF treated BMCs and this process was significantly inhibited by addition of Apt/PDGs^s@pMOF. h) Quantification results of MDSCs elimination in different groups. i) CCK‐8 assay of BMCs after treated with activators and various concentrations of different formulations for 48 h. Data are presented as means ± SD (*n* = 4). j) Flow cytometry analysis of p‐STAT3 expression of MDSCs with different treatments. k) Inhibition of STAT3 blocked its nuclear translocation in MDSCs, following by CLSM. G1: Control + L (laser irradiation), G2: Apt/PDGs^s@pMOF + L, G3: cApt/PDGs^s@pMOF + L, G4: Apt/PDG@pMOF + L, G5: Apt/PDGs^s@pMOF (P‐L, pre‐irradiated) + L, G6: Apt/PDGs^s@pMOF + D (dark). Significance is defined as ns, no significance, ^*^ *p* < 0.05, ^**^ *p* < 0.01, ^***^ *p* < 0.001.

We also tested the effect of Apt/PDGs^s@pMOF‐based PDT on ICD induction by measuring CRT exposure, HMGB1 release and ATP secretion.^[^ ^7^ ^]^ The CLSM results (**Figure** **3**a) showed that Apt/PDGs^s@pMOF significantly promoted CRT exposure on the surface of 4T1 cells (membrane staining with wheat grem agglutinin‐633 (WGA‐633)) and HMGB1 release from the nuclei upon incubation with a 5 min laser irradiation (G2), which was positively correlated with its highest uptake. Meanwhile, the flow cytometry results (Figure 3b) revealed that laser irradiation of Apt/PDGs^s@pMOF‐treated cells (G2) could induce over 15‐fold higher CRT exposure than that of light‐shielded group (G6), as well as increased HMGB1 release from 4T1 cells treated with Apt/PDGs^s@pMOF+L (laser irradiation) (G2) into extracellular fluid was detected (Figure 3c). Additionally, on account of ROS‐induced mitochondrial damage, the extracellular ATP secretion from 4T1 cells treated with Apt/PDGs^s@pMOF+L (G2) was significantly higher than that of others (Figure 3d). In summary, the above results verified Apt/PDGs^s@pMOF‐based PDT cumulatively inducing ICD of tumor cells in vitro.

We further evaluated the ICD‐meditated antitumor immunity by DC maturation investigation. The immature DCs were incubated with medium supernatants from several treated 4T1 cells for 48 h, and the frequency of matured DCs (CD11c^+^ CD80^+^ CD86^+^) was then analyzed via flow cytometry (Figure 3e). Compared with control group, PDT‐treatment groups showed a 2.15‐fold, 1.31‐fold, and 1.27‐fold increase in matured DC frequency, respectively. Particularly, the Apt/PDGs^s@pMOF +L group exhibited the highest DC maturation rate (nearly 20.5%).

MDSCs are a heterogeneous population of bone marrow‐derived immature cells that are recruited into tumor bed in tumorigenesis, infiltrating, and expansion, finally resulting in the inhibition of innate and adaptive immune responses via multiple T cell dysfunctions. Preventing MDSC recruitment, eliminating MDSCs, and inducing MDSC differentiation are the three major therapeutic strategies applied in MDSC modulation. Among them, MDSC elimination has been regarded as a more conventional way to target MDSCs both in basic and clinical studies.^[^ ^12^ ^]^ MDSCs are characterized as CD11b^+^ Gr‐1^+^ in mice, where the activation of STAT3 plays a critical role in the formation and expansion of MDSCs (Figure 3f). Firstly, we stimulated the bone marrow cells (BMCs) with interleukin‐6 (IL‐6) and granulocyte‐macrophage colony‐stimulating factor (GM‐CSF) to transform into MDSCs. As shown in Figure 3g, compared to control group, nearly 20‐fold increase in the frequency of CD11b^+^ Gr‐1^+^ MDSCs was measured in the activated group. Conversely, upon addition of Apt/PDGs^s@pMOFthe frequency of CD11b^+^ Gr‐1^+^ MDSCs was declined to 4.19%. Meanwhile, further investigation of the key components that played the suppressive role in MDSC formation showed that the PDG had a significantly higher inhibition effect than PD owing to the drug‐loading (Figure 3h), while the same‐low‐dose free GEM did not work at all in vitro (Figure S23, Supporting Information), indicating that the prodrug strategy could at least enhance the MDSC elimination capacity of GEM.

Cytotoxicity results showed that Apt/PDGs^s@pMOF could reduce the GEM‐induced myelosuppression (Figure 3i). Then, examination of STAT3 pathway in MDSCs was performed to ascertain the inhibition mechanism. Except for the PD‐treated group, all others showed the inhibition of STAT3 phosphorylation (Figure 3j), among which the pMOF, PDG and Apt/PDGs^s@pMOF exhibited the higher inhibition effect than free GEM. Additionally, the nuclear distribution of p‐STAT3 was significantly decreased with Apt/PDGs^s@pMOF treatment for 24 h (Figure 3k). Collectedly, we demonstrated that the designed Apt/PDGs^s@pMOF could specifically inhibit the formation and expansion of MDSCs via STAT3 pathway blockage.

### 2.4 Evaluation of Tumor Targeting and Penetration

**Figure 4:**
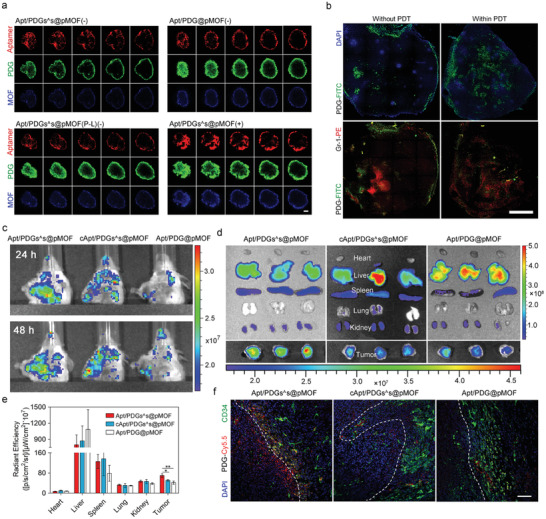
Evaluation of tumor targeting and penetration. a) Penetration efficiency of crosslinked Apt/PDGs^s@pMOF under several condition ((−): dark, (+): laser irradiation, (P‐L): pre‐irradiated) and non‐crosslinked Apt/PDG@pMOF into 4T1 tumor spheroids with 4 h of incubation and selective laser exposure for 5 min. The CLSM images were gained based on fluorescence signal of FITC‐labeled PDG, TRITC‐labeled Aptamer and pMOF. Scale bar: 75 µm. *Z*‐axis depth 20 µm. b) Penetration efficiency of FITC‐labeled PDG or Apt/PDGs^s@pMOF into tumor lesion in 4T1 tumor‐bearing mice with selective laser exposure for 10 min. Scale bar: 1 mm. c) In vivo images of tumor‐bearing mice intravenously administrated with Cy5.5‐labeled formulations at 24 and 48 h post the injection by IVIS. d) Ex vivo images of excised organs isolated from tumor‐bearing mice by IVIS at 48 h post the injection. e) Bio‐distribution of the Cy5.5‐labeled nanoparticles 48 h after intravenous injection into tumor‐bearing mice (*n* = 3). f) CD34‐staining and nanoparticle distribution in frozen tumor slices from tumor‐bearing mice at 48 h after administration with Cy5.5‐labeled nanoparticles. Scale bar: 75 µm. Significance is defined as ns, no significance, ^*^ *p* < 0.05, ^**^ *p* < 0.01, ^***^ *p* < 0.001.

Encouraged by the superior ICD induction and MDSC elimination in vitro, investigation of tumor targeting efficiency of Apt/PDGs^s@pMOF was followed up in the 4T1 breast tumor‐bearing mice by IVIS spectrum. Remarkably, the mice received Apt/PDGs^s@pMOF showed the strongest near‐infrared (NIR) signal at the tumor sites among the mice treated with dual targeting and crosslinked Apt/PDGs^s@pMOF, non‐targeting cApt/PDGs^s@pMOF and non‐crosslinked Apt/PDG@pMOF at 24 or 48 h after intravenous injection (**Figure** **4**c). In addition, the significantly higher NIR signal was also detected in ex vivo tumor in the Apt/PDGs^s@pMOF group (Figure 4d,e). Further CLSM imaging of tumor section showed that in the tumor regions distributed with the similar blood vessels (stained with CD34 antibody), the strongest Cyanine5.5 (Cy5.5) fluorescence of nanoparticles was measured in the Apt/PDGs^s@pMOF group, and the signal of Cy5.5 mainly distributed around blood vessels (Figure 4f). Summing up, the Apt/PDGs^s@pMOF showed an effective tumor‐targeting property, which was essential for subsequent antitumor therapy.

The natural physical barrier existing in solid cancer is still the challenge blocking the effective intratumoral delivery of most nanoparticles.^[^ ^17^ ^]^ Thus, we introduced a ROS‐sensitive crosslinker into our nanoparticle and hoped that when triggered by ROS, the crosslinkers would be cleaved, as well as the interaction force between PDG and pMOF would be reduced, which jointly resulted in the small‐size PDG escaping from the surface of pMOF to accomplish an enhanced tumor penetration. At this time, we first evaluated the penetration capability of Apt/PDGs^s@pMOF on tumor spheres. As shown in Figure 4a and Figure S27 in the Supporting Information, when incubated with Apt/PDGs^s@pMOF in dark for 4 h, the fluorescence of aptamer, PDG and MOF were only detected on the border of tumor sphere, suggesting the difficulty for Apt/PDGs^s@pMOF to achieve penetration as a whole, which might be associated with the size limitation (≈100 nm).^[^ ^17e^ ^]^ However, when treated with un‐crosslinked Apt/PDG@pMOF or pre‐irradiated Apt/PDGs^s@pMOF (P‐L), semiquantitative analysis showed that the PDG‐represented green fluorescence could reach deeper into the tumor spheroid while less accompanying shift found in aptamer and pMOF represented fluorescence (Figure S27, Supporting Information). These results revealed that PDG could penetrate into the deeper tumor lesion for its therapeutic effect playing once the crosslinking was interrupted. Otherwise, the aptamer, also with a small size, did not perform similar penetration behavior as PDG, which should be attributed to the influence by its negative charge and leaving after periostin‐binding. Notably, when irradiated at the time point of 1 h incubation and then incubated for another 3 h in dark, the tight physical barrier of tumor spheroids seemed to be cleaved by PDT, with the stronger infiltration of all three components, which might be associated with the degraded hyaluronic acid (HA) found upon PDT treatment under CLSM (Figure S28, Supporting Information), a major component of extracellular matrix. These results indicated that PDT could enhance the tumor penetration of Apt/PDGs^s@pMOF through crosslinking destruction and matrix degradation.

PDT‐triggered penetration of Apt/PDGs^s@pMOF was investigated in tumor‐bearing mice (Figure 4b). At 12 h after intravenous injection, the mice were received a laser irradiation or incubated shielded from light, and the irradiated or control mice were sacrificed after another 12 h incubation. When received PDT, the distribution of PDG‐represented green fluorescence tended to be more dispersed and infiltrated deeper into the tumor, as well as some of the dispersed PDG was co‐localized with the Gr‐1 antibody stained MDSC. In summary, all of the data validated the successful PDT‐triggered tumor deeper penetration of Apt/PDGs^s@pMOF.

### 2.5 Antitumor Immune Responses in Single 4T1 Tumor‐Bearing Mice Model

**Figure 5:**
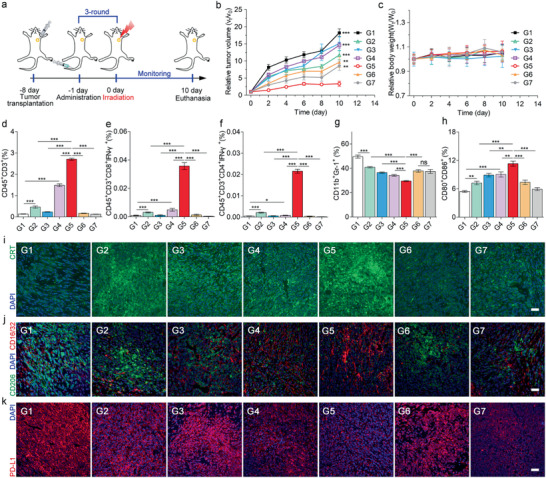
Antitumor immune responses in single 4T1 tumor‐bearing mice model. a) Treatment schedule for Apt/PDGs^s@pMOF‐mediated therapy. b) Tumor volume change and c) body weight change of 4T1 breast tumor‐bearing mice with different treatments. Data are presented as means ± SD (*n* = 5). d–f) CD3^+^ T cells, active CTLs, and Th1 cells levels in tumor lesions after various treatments, analyzed by flow cytometry (*n* = 3). g) MDSC levels in tumor lesions, analyzed by flow cytometry (*n* = 3). h) Matured DC levels in TDLN. i) CLSM examination of CRT exposure in different treated groups. Scale bar: 50 µm. j) Representative image increased M1 macrophages (stained with CD16/32) and declined M2 macrophages (stained with CD206). Scale bar: 50 µm. k) CLSM image of PD‐L1 expression. Scale bar: 50 µm. G1: Control + L (laser irradiation), G2: Apt/PDs^s@pMOF + L, G3: Apt/PDG@pMOF + L, G4: cApt/PDGs^s@pMOF + L, G5: Apt/PDGs^s@pMOF + L, G6: gemcitabine + L, G7: Apt/PDGs^s@pMOF + D (dark). Significance is defined as ns, no significance, ^*^ *p* < 0.05, ^**^ *p* < 0.01, ^***^ *p* < 0.001.

Subsequently, we assessed the therapeutic efficacy of Apt/PDGs^s@pMOF in the 4T1 tumor‐bearing BALB/c mice, following the therapeutic schedule (**Figure** **5**a). After the treatment (Figure 5b,c), we found that tumor‐targeting, prodrug‐loaded and crosslinked Apt/PDGs^s@pMOF+L (G5) had the best antitumor effect with no significant weight changes, suggesting that combination of the three strategies could maximize the pharmaceutical effect. As expected, the non‐crosslinked Apt/PDG@pMOF+L (G3) did not show tumor inhibition at all, which manifested the importance of crosslinking for effective delivery. Moreover, the non‐drug‐loaded Apt/PDs^s@pMOF+L (G2) and non‐targeting cApt/PDGs^s@pMOF+L (G4), due to the lack of GEM and weaker PDT effect respectively, just exhibited a moderate inhibition. Besides, the Terminal deoxynucleotidyl transferase dUTP nick‐end labeling (TUNEL) results (Figure S34, Supporting Information) also showed a significant cell apoptosis induced in Apt/PDGs^s@pMOF+L group (G5).

Then, we investigated whether the lacking immune cell infiltration in tumor area was improved after the treatment with formulations by flow cytometry and CLSM. As shown in Figure 5d and Figure S32 in the Supporting Information, the number of CD45^+^CD3^+^ T cells in control group (G1) and non‐PDT treated groups (G6 and G7) was in extremely low levels, which was in agreement with the initial report that TNBC was a “cold” tumor. Upon treated with PDT, there was a significant increase in CTL levels, indicating the successful promotion from “cold” tumor to “hot” tumor, among which the Apt/PDGs^s@pMOF+L (G5) played the most important role. Additionally, in Apt/PDGs^s@pMOF+L group (G5), a large number of CD8^+^ T cells infiltrated into deep tumor were present, while in the others only a spot of CD8^+^ T cells were found at the tumor boundary (Figure S35, Supporting Information). Further analysis of CTL and Th1 cells in tumor sites (Figure 5e,f) also proved the significant improvement of TILs in Apt/PDGs^s@pMOF+L group (G5).

The mechanism study of immune upregulation in treated mice was further carried out. Due to the active‐targeting accumulation, the most significant PDT‐induced CRT exposure was found in Apt/PDGs^s@pMOF + L (G5) and Apt/PDs^s@pMOF + L (G2) groups upon irradiation (Figure 5i). Then, as shown in Figure 5g and Figure S33 in the Supporting Information, all formulation‐treatments affected the tumor‐accumulation of MDSCs, which was consistent with MDSC elimination in vitro. In particular, the Apt/PDGs^s@pMOF + L group (G5) showed a nearly 40% decrease in the number of MDSCs while the drug‐unloaded group (G2) only had a 20% decrease, indicating that targeting prodrug delivery was beneficial for MDSC elimination in vivo. Meanwhile, though gemcitabine + L (G6) was responsible for the most significant decrease in MDSC distribution in spleen (Figure S36, Supporting Information), related to its rapid peripheral distribution, Apt/PDGs^s@pMOF +L (G5) also caused a significant decrease compared with control group, revealing that treatment with Apt/PDGs^s@pMOF +L (G5) could not only regulate the intratumoral MDSC distribution, but also played a crucial role in systemic MDSC regulation. DC maturation is a pivotal promoter for antigen presentation to T cells, thus enhancing intratumoral infiltration of CD8^+^ CTLs. Exposed CRT can promote DC phagocytosis and maturation, and MDSC elimination can relieve the direct inhibition of DCs by MDSCs.^[^ ^12^ ^]^ Therefore, compared with control group, the Apt/PDGs^s@pMOF +L (G5) efficiently facilitated the DC maturation (CD11c^+^ CD80^+^ CD86^+^) from 5.48% to 11.6% in tumor draining lymph nodes (TDLNs) (Figure 5h and Figure S31, Supporting Information), explaining the reason well why the Apt/PDGs^s@pMOF + L (G5) group had the most tumor‐infiltration levels of CTLs. Meanwhile, the significantly increased M1/M2 ratio of macrophage and declined PD‐L1 expression were certified with Apt/PDGs^s@pMOF + L (G5) treatment under CLSM (Figure 5j,k), which is also conducive for T cell immunity improvement.^[^ ^12^ ^]^ Moreover, the analysis of lymphocyte distribution in spleen showed that both the number of CD45^+^CD3^+^T cells and CD4^+^ or CD8^+^ subgroups were significantly upregulated in the Apt/PDGs^s@pMOF +L (G5) group, showing the mobilization of body immunity (Figure S37, Supporting Information).

### 2.6 Antitumor Immune Responses in Bilateral 4T1 Tumor Model

**Figure 6:**
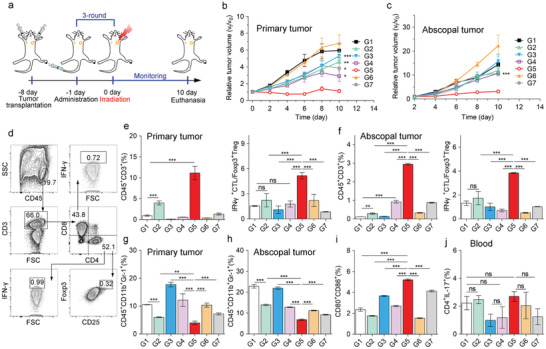
Antitumor immune responses in bilateral 4T1 tumor model. a) Treatment schedule for Apt/PDGs^s@pMOF‐mediated therapy in bilateral 4T1 tumor model. b) Primary tumor volume change and c) abscopal tumor volume change in BALB/c mice. Data are presented as means ± SD (*n* = 5). d) Gating strategy to determine frequencies of TILs from tumor lesions. e) CD3^+^ T cells and CTL/Treg levels in primary tumor lesions and f) abscopal tumor lesions, analyzed by flow cytometry (*n* = 3). g) MDSCs levels in primary tumor lesions and h) abscopal tumor lesions, analyzed by flow cytometry (*n* = 3). i) Matured DC levels in TDLN, analyzed by flow cytometry (*n* = 3). j) Th 17 T cells levels in blood of tumor‐bearing mice, analyzed by flow cytometry (*n* = 3). G1: Control + L (laser irradiation), G2: Apt/PDs^s@pMOF + L, G3: Apt/PDG@pMOF + L, G4: cApt/PDGs^s@pMOF + L, G5: Apt/PDGs^s@pMOF + L, G6: gemcitabine + L, G7: Apt/PDGs^s@pMOF + D (dark). Significance is defined as ns, no significance, ^*^ *p* < 0.05, ^**^ *p* < 0.01, ^***^ *p* < 0.001.

To investigate the therapeutic effect of enhanced body immunity, a bilateral 4T1 tumor model was established as described. The primary tumor was treated with the same therapeutic schedule previously mentioned and the abscopal tumor without any extra‐treatment (**Figure** **6**a). Similar to the treatment results obtained on single 4T1 tumor‐bearing mice model, tumor proliferation on the primary side was inhibited in Apt/PDGs^s@pMOF + L (G5) group (Figure 6b and Figure S29, Supporting Information). Due to the upregulated body immunity, the growth of abscopal tumor was significantly inhibited in Apt/PDGs^s@pMOF + L (G5) group (Figure 6c). Further verification of immune status in tumors showed the amount of CD45^+^ CD3^+^ TILs was increased on both sides upon Apt/PDGs^s@pMOF + L (G5) treatment, especially the proportion of CTLs/Tregs was significantly improved (Figure 6e,f). Similarly, MDSC was clearly downregulated on both sides after Apt/PDGs^s@pMOF + L (G5) treatment (Figure 6g,h), as well as the number of mature DC was also increased within this treatment (Figure 6i). Additionally, there were more CD3^+^ CD8^+^ and CD3^+^ CD4^+^ T cells in blood in Apt/PDGs^s@pMOF + L (G5) group (Figure S38, Supporting Information), forcefully indicating the upregulation of body immunity. However in fact, the body immune upregulation is often considered to be prone to irAEs, and IL‐17^+^ CD4^+^ Th17 are usually highly upregulated in inflammatory tissues of autoimmune diseases, suggesting the ratio of Th17 cells a parameter for irAEs surveillance of immunotherapy.^[^ ^3c^ ^]^ As shown in Figure 6j, although the amount of Th17 cells were observed with 1.2‐fold increase in Apt/PDGs^s@pMOF + L (G5) group, which has been reported to be conducive to antitumor immunity, there was no significant difference between Apt/PDGs^s@pMOF + L (G5) group and control group (G1). These results manifested the advantages of the Apt/PDGs^s@pMOF formulation we designed, which could significantly enhance the antitumor immunotherapy effect in local tumor lesions within less severe irAEs in the body, providing a promising therapeutic strategy for anti‐TNBC immunotherapy. Importantly, as shown in Figure S39 in the Supporting Information, the negligible change in the concentrations of alanine transaminase (ALT), aspartate transaminase (AST), serum albumin (ALB) and alkaline phosphatase (ALP) suggested negligible liver toxicity of Apt/PDGs^s@pMOF. Similarly, inappreciable variations in the populations/concentrations of red blood cell (RBC), white blood cell (WBC), platelets (PLT), hemoglobin (HGB) also indicated the less cytotoxicity of Apt/PDGs^s@pMOF in vivo. Furthermore, H&E staining images of major organ sections excised from mice treated with multiple formulations indicated the biosafety of the pMOF‐based nanoparticles (Figure S40, Supporting Information).

## 3 Conclusion

In summary, we reported a novel layer‐by‐layer Apt/PDGs_^_s@pMOF nanoplatform to reinforce the intratumoral infiltration of CTLs and reeducate the immunosuppressive TIME, synergistically contributing to effective antitumor immunotherapy. Particularly, Apt/PDGs_^_s@pMOF could accomplish valid tumor accumulation and penetration via aptamer‐mediated active‐targeting and PDT‐triggered PDG release, respectively. The pMOF‐based PDT facilitated ICD response for DC maturation and further CTL infiltration, and the penetrated PDG could selectively eliminate intratumoral MDSCs to reverse immunosuppressive TIME. Hence, Apt/PDGs_^_s@pMOF+L could elevate specific antitumor T‐cell responses in both primary and abscopal tumors in the bilateral 4T1 model and lead to growth inhibition. Finally, these above advantages enable Apt/PDGs_^_s@pMOF a promising strategy for anti‐TNBC immunotherapy.

## 4 Experimental Section

##### Materials

*α*‐Methoxy‐*ω*‐succinimidyl carboxymethyl ester‐poly (ethylene glycol) (CH_3_O‐PEG‐NHS, Mw 5000) was purchased from Jenkem Technology (Beijing, China). Pyrrole, 4‐carboxybenzaldehyde, 6‐maleimidohexanoic acid, 3‐mercaptopropionic acid, and *N*‐hydroxysulfosuccinimide sodium salt were purchased from J&K Chemical Ltd. (Shanghai, China). 2‐Iminothiolane hydrochloride was purchased from Aladdin (Shanghai, China), Zirconium (IV) oxychloride octahydrate, tert‐butylchlorodimethylsilane, tetrabutylammonium fluoride and fluorescein isothiocyanate (FITC) were purchased from Energy Chemical (Shanghai, China). Gemcitabine, cell counting kit‐8 and various dialysis bags were purchased from Dalian Meilun Biotechnology Co., Ltd. (Dalian, China). Dendri‐graft‐l‐lysine (G3, Mw 22 000) was purchased from Colcom (Clapiers, France). Periostin‐targeting aptamer and negative control (PNDA‐3: ACGAGYYGYCGCAYGYGCGGYYCAGYCYGGYCCYYCAGCACCGYACAACAA; negative control: ACGAGYCACACGYYGAYGACYGGAYGGYAGYYAAAGAGGGYGGGGCAACAA) was synthesized by Genscript (Nanjing, China). 2′,7′‐Dichlorofluorescein diacetate (H_2_DCFH‐DA) and 4′,6‐diamidino‐2‐phenylindole (DAPI) were purchased from Sigma‐Aldrich (St. Louis, USA). Hoechst 33342 and LysoTracker DND Green were purchased from Molecular Probes (Waltham, USA). Cy5.5‐NHS (684/710 nm) was purchased from APExBIO (Houston, USA). Terminal deoxynucleotidyl transferase dUTP nick‐end labeling (TUNEL) assay and annexin V‐FITC/PI apoptosis detection kit were purchased from KeyGEN Biotech Co., Ltd. (Nanjing, China). Enhanced ATP assay kit was purchased from Beyotime Biotechnology (Shanghai, China). All the other chemical reagents were purchased from Sinopharm Chemical Reagent Co., Ltd. (Shanghai, China).

##### Cell Lines

4T1 cells were cultured in RPMI 1640 medium supplemented with 10% fetal bovine serum, 10 U mL^−1^ penicillin and 10 µg mL^−1^ streptomycin at 37  °C and 5% CO_2_ in a humidified atmosphere.

##### Mouse Model Establishment

Female BALB/c mice of ≫20 g body weight were obtained from Department of Experimental Animals (Fudan University) and maintained under standard laboratory conditions. All animal handling procedures were approved by Institutional Animal Care and Use Committee of China (2019‐03‐YJ‐JC‐01). The single orthotopic 4T1 breast tumor model was established by injection of 1.0  ×  10^6^ 4T1 cells in 100 µL PBS 7.4 into the second breast pad of female BALB/c mice. The bilateral 4T1 tumor model was established by orthotopically injecting 1.0  ×  10^6^ 4T1 cells into the left second breast pad and 2.0  ×  10^5^ 4T1 cells into the right second breast pad of the female BALB/c mice, which were designated as the primary and the abscopal tumors, respectively.

##### Synthesis of Porphyrin, PEG‐DGL‐GEM Polymer, and ROS‐Sensitive Linker

All the compounds were synthesized according to designed routes (Scheme S1, Supporting Information). Detailed process and characterization are presented in the Supporting Information.

##### Synthesis of pMOF Nanoparticles

pMOF was synthesized according to the reported protocol. Briefly, TCPP (50 mg), ZrOCl_2_·8H_2_O (150 mg), and benzoic acid (1.4 g) were dissolved in DMF (60 mL) and stirred for 5 h at 90 °C. After this, the obtained mixture was centrifuged at 14 000 rpm for 60 min. The obtained particles were thoroughly washed with DMF three times and dd‐water three times. The obtained pMOF particles were stored in dd‐water shielded from light.

##### Preparation of Apt/PDGs_^_s@pMOF

Apt/PDGs_^_s@pMOF was prepared via a lay‐by‐lay method. In detail, 10 µL PDG aqueous solution (5 mg mL^−1^) was added into 100 µL pMOF aqueous solution (1.5 mg mL^−1^) under an ultrasonic dispersion and a dispersive solution of PDG @pMOF was acquired. Next, 30 eq NHS‐activated crosslinkers were added into the obtained PDG @pMOF solution and sealed in a dialysis bag (diameter  =  40 mm, MWCO  =  20 000) followed by dialysis against deionized water for 48 h to form the PDGs_^_s@pMOF. Then, 15 µL Aptamer aqueous solution (10 mg mL^−1^) was added into the obtained MSDG solution to gain the final formulation (Apt/PDGs_^_s@pMOF).

Sizes and Zeta potential of the freshly prepared nanoparticles were measured in deionized water by dynamic light scattering (DLS) (Zetasizer Nano‐ZS, Malvern, U.K.). The morphological images of the formulations were photographed by transmission electron microscope (TEM, Tecnai G2 spirit Biotwin, FEI). HAADF images and the distribution for the elemental mapping of Zr, C, N, O, S, F, P of Apt/PDGs_^_s@pMOF and Apt/PDs_^_s@pMOF were photographed by Field Emission Transmission Electron Microscope (FE‐TEM, Talos F200X, FEI). Emission spectra excited at 405 nm of pMOF before and after capped with PDG and Aptamer were measured by Agilent Technologies Cary Eclipse Fluorescence Spectrophotometer. Furthermore, the stability test of nanoparticles was carried out in DMEM (1% FBS) for 7‐day incubation, and the size and zeta potential of the nanoparticle were measured every day by DLS.

##### Hemolysis Evaluation

RBC were diluted to isotonic working solution with fixed concentration (10^6^ cells mL^−1^). Different samples (800 µL) with a series of concentrations (6.25, 12.5, 25, 50, 100 and 200 µg mL^−1^) dissolved in isotonic working solution were added with RBC suspension (200 µL) and incubated for 2 h at 37 °C with gentle shaking (30 rpm). Then, the samples were centrifuged (1700 rpm, 5 min, 4 °C) to isolate the supernatant from RBC. The supernatant (200 µL) was transferred to a 96‐well plate, and the absorbance at 541 nm was determined on a Multiskan MK3 microplate reader (Thermo Scientific, Waltham, MA, USA) with absorbance at 655 nm as the references. RBC suspension (200 µL) added with isotonic working solution (800 µL) and treated through the same procedure was used as the negative control. RBC suspension (200 µL) directly added with distilled water (800 µL) and treated through the same procedure was used as the positive control. The hemolysis percentage was calculated by the following equation: Hemolysis percentage = ((each sample's absorbance‐negative control absorbance)/(positive control absorbance‐negative control absorbance))∗100%

##### Singlet Oxygen Generation

A 30 mW cm^−2^ 660 nm LED light was used to generate singlet oxygen and 2′,7′‐dichlorodihydrofluorescein diacetate (H_2_DCFH‐DA) was employed to monitor singlet oxygen generation. 2.44 mg of H_2_DCFH‐DA was diluted in 1 mL of DMSO to achieve a final concentration of 5 × 10^−3^ m H_2_DCFH‐DA stock. Adding 100 µL of H_2_DCFH‐DA stock into pMOF (8 µg mL^−1^, 10 mL) solution and starting irradiation. During this time, taking out 500 µL of reaction mixture for fluorescence measurement at setting time point. The H_2_DCFH‐DA was excited at 485 nm, and the emission between 485–600 nm was read (ex/em slits = 5.0/10.0 nm, PMT voltage = 700 V). Fluorescence was measured after the irradiation at 485 nm for 0, 5, 10, 15, 20, 25, 30, 35, and 40 min.

To determine the appropriate dose concentration of pMOF for further treatment, keeping the irradiation time at 5 min and investigating the ROS generation responded to serial concentrations of pMOF. Briefly, 200 µL serial concentrations of pMOF (2–32 µg mL^−1^) was added into 96‐well black plate which has been pre‐incubated with 2 µL H_2_DCFH‐DA stock solution (50 × 10^−3^ m). After irradiation for 5 min, the fluorescence intensity was measured by Multiskan MK3 microplate reader at *λ*_ex/em_, 485 nm/528 nm.

##### In Vitro Drug Release Study

Dendrimer PDGs were dissolved in PBS 7.4 to form a final gem concentration of 50 µg mL^−1^ PDG solutions, and the solution was equally divided into 30 ep tubes, experiment was operated in a shaking bath at 100 rpm, 37 °C. At the time point of 0, 1, 2, 4, 8, 12, 24, 48, 72, 96 h, randomly selecting 3 samples for gemcitabine contents detection by HPLC, measured by UV detector at 268 nm (10% CH_3_OH, 90% H_2_O).

##### Intracellular Gemcitabine Release Study

4T1 cells were seeded into 24‐well plate (Wuxi NEST Biotechnology Co., Ltd. Wuxi, China) with a density of 1.0 × 10^5^ cells per well and cultured at 37 °C for 12 h. Cells were assigned to six groups, and cells in each group were treated with 100 µL FITC labeled PDG‐FITC (1.32 mg mL^−1^) solution at the set time point of 0, 1, 2, 3, 4, 5 h. Then, at the 6 h, all cells were rinsed with Hank's for three times and added 300 µL internal standard (cefaclor, 20 ng mL^−1^) in PBS 7.4. After a seven times repeated freezing and thawing in liquid nitrogen, the cells and medium solution was collected and centrifuged at 3000 rpm for 5 min. Taking 150 µL from per sample and adding it to the 96‐well blackboard for FITC detection by using a Multiskan MK3 microplate reader (Thermo Scientific, Waltham, Massachusetts, USA). Taking 100 µL from per sample and adding 100 µL methanol to precipitate salt and proteins for further gemcitabine measurement via LC–MS.

##### Cell Uptake Study

4T1 cells were seeded in a 48‐well plate (Corning‐Coaster, Tokyo, Japan) at a density of 3  ×  10^4^ cells per well. When achieving 80–90% confluence, the cells were incubated with FITC‐labeled Apt/PDGs_^_s@pMOF, cApt/PDGs_^_s@pMOF, Apt/PDG@pMOF and Apt/PDGs_^_s@pMOF (pre‐irradiated for 5 min) in serum‐free RPMI for 60 min. Then the cells were rinsed with Hank's for three times. The cellular uptake of nanoparticles was visualized and photographed by Confocal Laser Scanning Microscope (CLSM, Carl Zeiss LSM710, Carl Zeiss, Jena, Germany) and quantified by flow cytometer (FACS, BD Biosciences, Bedford, MA). As for investigating the cellular internalization mechanism of the Apt/PDGs_^_s@pMOF, aptamer was labeled with TRITC, PDG was labeled with FITC. 4 °C, 1 µg mL^−1^ colchicine, 0.2 µg mL^−1^ phenylarine oxide (PhAsO) and 0.4 µg mL^−1^ filipin complex treated cells as endocytic inhibitors for energy‐dependent, macropinocytosis, clathrin, and caveolin pathway, respectively.

Intracellular distribution of Apt/PDGs_^_s@pMOF: To investigate the intracellular distribution of Apt/PDGs_^_s@pMOF, immunofluorescence study was performed to visualize the lysosome in cells treated with Apt/PDGs_^_s@pMOF. 4T1 cells were seeded in 35 mm confocal dishes (5  ×  10^3^ cells per well). After 12 h incubation, cells were treated with FITC, TRITC‐labeled Apt/PDGs_^_s@pMOF for 1 or 3 h, and then the cells were rinsed with Hank's for three times. Afterward, the cells were stained with LysoTracker Deep Red to visualize the lysosome, and the cell nuclei were stained with DAPI. The intracellular distribution of Apt/PDGs_^_s@pMOF was visualized and photographed by CLSM.

##### In Vitro and In Vivo Tumor‐Penetration Study

For in vitro penetration study, a tumor spheroid model was established as previously reported. Firstly, 150 µL per well DMEM (containing 2% agarose) was added to 24‐well plates and cold to form a supporting layer. Then, 4T1 cells were seeded over the agarose layer at a density of 4000 cells per well for 5 day and the tumor spheroids of about 200 µm in diameter were obtained. The tumor spheroids were incubated with Apt/PDGs_^_s@pMOF, Apt/PDG@pMOF, Apt/PDGs_^_s@pMOF (pre‐irradiated for 5 min) which were composed of FITC‐labeled PDG and TRITC‐labeled Aptamer for 4 h in the dark. At the first 1 h, the spheroids treated with Apt/PDGs_^_s@pMOF were irradiated for 5 × 1 min with 1 min interval (660 nm LED light, 30 mW cm^−2^) or incubated shielded from light. Finally, the tumor spheroids were washed, fixed, and transferred to 35 mm confocal dishes for observation under confocal fluorescence microscope.

For in vivo penetration study, FITC‐labeled Apt/PDGs_^_s@pMOF were intravenous injected to the 4T1 tumor‐bearing mice to evaluate irradiation‐triggered penetration efficiency in solid tumor. 24 h after injection, the mice were irradiated for 5 min (660 nm laser, 30 mW cm^−2^) or incubated shielded from light (control), and irradiated or control mice were sacrificed 12 h after treatment. Tumor tissue was excised after perfusion and fixed in 4% neutral paraformaldehyde. Cryo‐x sections of 10 µm were made followed by staining with PE‐labeled Gr‐1 antibody and DAPI to demonstrate MDSC and nucleus. The sections were then observed and photographed under fluorescence

##### Measurement of Intracellular ROS Generation

4T1 cells were seeded into 96‐well black plates with a density of 5 × 10^4^ cells per well and cultured at 37 °C for 12 h. Cells were treated with Apt/PDGs_^_s@pMOF, cApt/PDGs_^_s@pMOF, Apt/PDG@pMOF, Apt/PDGs_^_s@pMOF (pre‐irradiated) at an equal pMOF concentration of 8 µg mL^−1^ for 1 h, and 5 × 10^−3^ m ROS scavenger, *N*‐acetyl‐l‐cysteine (NAC) was used as an inhibited control. After being rinsed with Hank's for three times, 100 µL H_2_DCFH‐DA were added at concentration of 20 × 10^−6^ m and the cells were cultured in 5% CO_2_ for 2 h. Afterward, the cells were incubated with irradiation (660 nm LED light, 30 mW cm^−2^) or shielded from light for 5 min. Then, the fluorescence intensity was measured by Multiskan MK3 microplate reader at *λ*_ex/em_, 485 nm/528 nm.

##### Cytotoxicity Assay

The cytotoxicity was evaluated by CCK‐8 assay. Briefly, 4T1 cells were seeded in 96‐well plates (NEST Biotechnology, Wuxi, China) at a density of 5000 cells per well and cultured in 5% CO_2_ at 37 °C for 12 h. Then 200 µL gradient concentrations of pMOF, Apt/PDs_^_s@pMOF, Apt/PDGs_^_s@pMOF dissolved culture medium was added to the 96‐well plates. After incubation for 1 h, the cells were irradiated for 5 min (660 nm LED light, 30 mW cm^−2^) or incubated in dark. After another 48 h incubation in dark, the culture media was removed and 200 µL CCK‐8 solution in PBS 7.4 was added, and the plate was incubated at 37 °C for 4 h before detected at 450 nm by using a Multiskan MK3 microplate reader (Thermo Scientific, Waltham, Massachusetts, USA). Cell viability was calculated as the survival percentage of control.

Moreover, the cytotoxicity was also evaluated by CLSM. After incubation with formulations at an equal pMOF concentration of 8 µg mL^−1^ for 1 h, the cells were irradiated for 5 min or incubated in the dark. Then, the cells were incubated in dark for another 1 h, and after that the cells were stained with annexin V‐FITC and PI and analyzed by CLSM.

##### Detection of CRT on the Cell Surface

4T1 cells were seeded into 35 mm confocal dishes at a density of 1 × 10^5^ cells per well and cultured at 37 °C for 12 h. After treated with the indicated agents for 1 h, cells were washed thrice with cold Hank's and another 200 µL Hank's were added. Then, the cells were exposed to 660 nm LED light (30 mW cm^−2^) or shielded from light for 5 min, and incubated for another 1 h. After that, the cells were washed again and fixed in 0.1% paraformaldehyde in PBS for 5 min at 0 °C. Then the cells were washed thrice in cold Hank's, and CRT antibody (diluted in cold blocking buffer) was added for 1 h. After three washes in cold Hank's, the cells were incubated with the Alexa 488‐conjugated secondary antibody and WGA‐630 (for cell surface staining) for another 1 h. In the end, the cells were fixed with 4% paraformaldehyde for 10 min before nuclear staining with DAPI (1/1000) in cold PBS. The CRT exposure was visualized and photographed by CLSM.

In addition, the cells were also collected for flow cytometric analysis of CRT exposure. After collected and washed thrice, living cells were incubated with CRT primary antibody (diluted in cold blocking buffer which contains 2% fetal bovine serum) on the ice for 1 h, followed by washing and incubation with the appropriate Alexa 488‐conjugated monoclonal secondary antibody in blocking buffer (for 1 h). Each sample was analyzed by flow cytometry to identify cell surface CRT. Antibodies used are listed in Table S3 in the Supporting Information.

##### HMGB1 Release Assay

Cells were seeded into 35 mm confocal dishes at a density of 1 × 10^5^ cells per well and cultured at 37 °C for 12 h. After treated with the same procedure mentioned in CRT detection, the cells were rinsed three times with cold Hank's, fixed in 4% paraformaldehyde for 10 min, permeabilized with 0.1% Triton X‐100 for 10 min and washed thrice with cold Hank's. After blocked with 10% fetal bovine serum in Hank's for 30 min, HMGB1 primary antibody was added for 1 h. Subsequently, the cells were washed again, incubated with an Alexa Fluor 488‐conjugated secondary antibody and WGA‐630 (for cell surface staining) for 1 h, and stained with DAPI (1/1000) in cold PBS. The HMGB1 release was visualized and photographed by CLSM.

Moreover, the HMGB1 release was also measured by western blot. After the same treatment, the supernatants were collected, dying cells were removed by centrifugation, and supernatants were concentrated to 100 µL by ultrafiltration. The adhesive cells were lysed with RIPA lysis buffer and centrifugated to form the purified membrane vesicles samples. Finally, the concentrated supernatants and cell samples were to resolved on SDS‐PAGE and analyzed by immunoblotting using HMGB1 and *β*‐actin antibodies, followed by enhanced chemiluminescence (ECL) detection (Millipore, Darmstadt, Germany).

##### ATP Secretion Assay

4T1 cells were seeded in 48‐well plates at a density of 1 × 10^5^ cells per well and cultured in 5% CO_2_ at 37 °C for 12 h. After treated with the same procedure mentioned in CRT detection, the supernatants were collected and dying cells were removed by centrifugation. 50 µL supernatants was used for ATP assay following by the manufacture's instruction.

##### Immunofluorescence

For section staining, first, to remove the embedding medium, the frozen tumor sections were washed with PBS 7.4 for 10 min × 3. The sections were incubated at 0 °C overnight with a primary antibody or fluorescence‐conjugated primary antibody, diluted in blocking buffer (2% fetal bovine serum and 0.5% Triton X‐100), and then rinsed three times with PBS. Afterward, the specimens were incubated with a fluorescence‐labeled secondary antibody or PBS for 2 h in the dark. Before detection by CLSM, the nucleus was stained with DAPI and then washed thrice with PBS for 10 min × 3. Antibodies used in immunofluorescence are listed in Table S3 in the Supporting Information.

##### DCs Maturation In Vitro

Tibias and femurs from healthy BALB/c mice were removed via sterile techniques and bone marrow cells (BMCs) were flushed. After passed through a 70 µm cell strainer to remove small pieces of muscle and bone, BMCs were resuspended in RPMI 1640 medium, containing 15% FBS for 24 h. Then, the adherent cells were cultured for 4 days in RPMI 1640 medium, containing 15% FBS, 10 ng mL^−1^ GM‐CSF (Sigma) and 10 ng mL^−1^ IL‐4 (Sigma). On day 5, DCs were seeded into 24‐well plates at a density of 2 × 10^5^ cells per well, and the medium was replaced by conditioned medium of 4T1 cells (cells were treated with the same process mentioned in the study of HMGB1 release) for 24 h. After that, flow cytometric analysis of mature DCs following staining. Fluorescence conjugated antibodies used in flow cytometry are listed in Table S1 in the Supporting Information.

##### MDSC Inhibition

Tibias and femurs from healthy BALB/c mice were removed via sterile techniques and BMCs were flushed. After passed through a 70 µm cell strainer to remove small pieces of muscle and bone, 2 × 10^6^ cells per well BMCs were plated into 24 well plates in 400 µL of medium supplemented with 40 ng mL^−1^ GM‐CSF and 40 ng mL^−1^ IL‐6. Cells were maintained at 37 °C in 5% CO_2_‐humidified atmosphere for 3 days. several formulations were added to the culture at day 0, following the concentrations: [Gem] 0.4 µg mL^−1^, [pMOF] 20 µg mL^−1^. After that, flow cytometric analysis of MDSC following staining.

As for p‐STAT3 detection, the formulations were added to the culture at day 3 when MDSCs have been activated by IL‐6 and GM‐SCF, with the same treatment concentrations. Then, flow cytometric analysis of p‐STAT3 expression in MDSC following staining. Fluorescence conjugated antibodies used in flow cytometry are listed in Table S3 in the Supporting Information.

In addition, the inhibition of p‐STAT3 expression was also investigated by immunofluorescence and photographed by CLSM. Antibodies used in immunofluorescence are listed in Table S3 in the Supporting Information.

##### Cytotoxicity of BMCs Assay

BMCs were seeded in 96‐well plates (NEST Biotechnology, Wuxi, China) at a density of 5000 cells per well and cultured in 5% CO_2_ at 37 °C for 12 h. Then 200 µL gradient concentrations of Apt/PDGs_^_s@pMOF and gemcitabine dissolved culture medium was added to the 96‐well plates. After 48 h incubation in dark, the culture media was removed and 200 µL CCK‐8 solution in PBS 7.4 was added, and the plate was incubated at 37 °C for 4 h before detected at 450 nm by using a Multiskan MK3 microplate reader (Thermo Scientific, Waltham, Massachusetts, USA). Cell viability was calculated as the survival percentage of control.

##### In Vivo Imaging and Biodistribution Studies

Cy5.5‐labeled Apt/PDGs_^_s@pMOF, cApt/PDGs_^_s@pMOF and Apt/PDG@pMOF were injected intravenously via tail vein into 4T1 breast tumor cells‐bearing mice at a dose of 0.1 mg Cy5.5 (684/710 nm) kg^−1^. The in vivo biodistribution of nanoparticles at the time point was traced and visualized by Xenogen IVIS Spectrum CT (Perkin Elmer Inc., Waltham, Massachusetts, USA). At 48 h after injection, mice were sacrificed. The ex vivo biodistribution of Cy5.5‐labeled nanoparticles was also assessed and semiquantified by IVIS.

##### Intratumoral Distribution Study

After injected with Cy5.5‐labeled Apt/PDGs_^_s@pMOF, cApt/PDGs_^_s@pMOF and Apt/PDG@pMOF for 48 h, the mice were sacrificed. The frozen tumor sections were stained with CD34 antibody and an Alexa Fluor 488‐conjugated secondary antibody to visualize the blood vessels, and the nuclei were stained with DAPI. The fluorescence was observed under CLSM. Antibodies used in immunofluorescence are listed in Table S3 in the Supporting Information.

##### Antitumor Efficacy

In vivo: According to the tumor size, female BALB/c mice with 4T1 breast tumor xenograft were randomly divided into seven groups (*n* = 5). The mice were administrated with saline (G1), Apt/PDs_^_s@pMOF (G2), Apt/PDG@pMOF (G3), cApt/PDGs^s@pMOF (G4), Apt/PDGs^s@pMOF (G5), gemcitabine (G6), Apt/PDGs^s@pMOF (G7) (with an equal dose of 10 mg kg^−1^ pMOF or an equal dose of 200 µg kg^−1^ gemcitabine) at day 7, 10, 13 post implantation and irradiated for 5 min (660 nm laser, 300 mW cm^−2^) or incubated shielded from light at day 8, 11, 14 post implantation. Tumor volume and body weight were recorded every other day. Tumor volume (*V*_t_) was calculated according to the following equation: *V*_t_  =  *a*  ×  *b* ^2^/2, where a is the longest and b is the shortest axes of the tumor in mm. Following the request of Fudan University Ethics Committee, the survival rate of the mice was not evaluated, since TNBC‐bearing mice are not fatal and the death is mainly caused by the hindered food intake. When the tumor volume of control group (G1) was over 1000 mm^3^, all the treated mice were sacrificed. Tumors were isolated from the mice and snap‐frozen to −80 °C in optimal cutting medium (O.C.T.). The frozen tumor tissue sections were sliced by freezing microtome and mounted on slides. TUNEL assay was performed on the obtained frozen tumor slices by using a One Step TUNEL Apoptosis Assay Kit, following by manufacture’ protocol. The stained tumor slices were examined by CLSM.

##### Flow Cytometry Assay of Immune Cells Population

Tumor draining lymph nodes (TDLN), tumor tissues, leukocytes in blood, spleen were dispersed into single‐cell suspensions. Then the TDLN cells, tumor‐infiltrating lymphocytes, leukocytes in blood and splenocytes were quantitatively analyzed by flow cytometry following by immunofluorescence staining. Briefly, tissues were harvested and dispersed into single‐cell suspensions by using 70 µm cell strainers (BD Pharmingen, New Jersey, America), and then adding 5 mL red blood cell lysis buffer into the suspensions to lysis the red blood cells. After that, cells were collected and dispersed into 200 µL PBS and 10 µL 1% BSA was added into suspensions to block the nonspecific binding. Then, staining the cells following the intracellular antigens detection protocols provided by Thermo Fisher, and analyzing cells by flow cytometry took place. Fluorescence conjugated antibodies used in flow cytometry are listed in Table S3 in the Supporting Information.

##### *In Vivo* Toxicity of Apt/PDGs^s@pMOF

The 12‐week old female BALB/c mice were administrated (i.v.) with Apt/PDGs^s@pMOF and gemcitabine (with an equal dose of 200 µg kg^−1^ gemcitabine) every four days for three times, and the saline was administrated as the control. After treatment, the mice were sacrificed, and the concentrations of red blood cell (RBC), white blood cell (WBC), platelets (PLT), hemoglobin (HGB), alanine transaminase (ALT), aspartate transaminase (AST), alkaline phosphatase (ALP) and serum albumin (ALB) in the blood were evaluated by a third‐party company (Wuhan Servicebio Technology Co., Ltd, Wuhan, China). The results are detailed in Figure S39 in the Supporting Information.

##### H&E Staining

The main organs (heart, liver, spleen, lung, and kidney) of the mice received one‐course treatments with different formulations were harvested and fixed with 4% paraformaldehyde. After routinely paraffin embedding, the organs were sectioned into 10 µm pieces, stained with hematoxylin and eosin, and finally photographed by digital microscopy.

##### Statistical Analysis

All the data were presented as means ± standard deviation (SD), and comparison between groups were performed by unpaired t‐test. Statistical significance was defined as ns, no significance, ^*^ *p* < 0.05, ^**^ *p* < 0.01, ^***^ *p* < 0.001.

## Conflict of Interest

The authors declare no conflict of interest.

## Supporting information

Supporting Information
