# Supplementary material for: Penetrable Nanoplatform for “Cold” Tumor Immune Microenvironment Reeducation
Source: Adv Sci (Weinh). 2020 Jul 29;7(17):2000411. doi: 10.1002/advs.202000411 (PMC7503208; doi:10.1002/advs.202000411)
Supplement: Supplementary file 1 — Supporting Information [file ADVS-7-2000411-s001.pdf]

Copyright WILEY-VCH Verlag GmbH & Co. KGaA, 69469 Weinheim, Germany, 2020.

ADVANCED SCIENCE
Open Access

Supporting Information

for *Adv. Sci.*, DOI: 10.1002/advs.202000411

Penetrable Nanoplatform for "Cold" Tumor Immune Microenvironment Reeducation

*Qinjun Chen, Yongqing He, Yu Wang, Chao Li, Yujie Zhang, Qin Guo, Yiwen Zhang, Yongchao Chu, Peixin Liu, Hongyi Chen, Zheng Zhou, Wenxi Zhou, Zhenhao Zhao, Xiaomin Li, Tao Sun, and Chen Jiang**

Supporting Information

Penetrable Nanoplatform for “Cold” Tumor Immune Microenvironment Reeducation

*Qinjun Chen, Yongqing He, Yu Wang, Chao Li, Yujie Zhang, Qin Guo, Yiwen Zhang, Yongchao Chu, Peixin Liu, Hongyi Chen, Zheng Zhou, Wenxi Zhou, Zhenhao Zhao, Xiaomin Li, Tao Sun, and Chen Jiang**

Q. Chen, Y. He, Y. Wang, C. Li, Y. Zhang, Q. Guo, Y. Zhang, Y. Chu, P. Liu, H. Chen, Z. Zhou, W. Zhou, Z. Zhao, Prof. T. Sun, Prof. C. Jiang
Key Laboratory of Smart Drug Delivery (Ministry of Education)
State Key Laboratory of Medical Neurobiology and MOE Frontiers Center for Brain Science
Institutes of Brain Science
Department of Pharmaceutics
School of Pharmacy
Research Center on Aging and Medicine
Fudan University
Shanghai 201203, People's Republic of China
E-mail: jiangchen@shmu.edu.cn
Prof. X. Li
Department of Chemistry and Laboratory of Advanced Materials
Fudan University
Shanghai 200433, People's Republic of China

Contents:

Scheme S1. Synthesis route of porphyrin, PEG-DGL-GEM polymer and ROS-sensitive linker

Figure S1-S12. ^{1}H NMR, MS-ESI, and GPC characterization of compounds

Figure S13. Formulation optimization of nanoplatforms

Figure S14. DLS histogram data of nanoplatforms

Figure S15. Hemolysis of RBCs

Figure S16. PDT effects and drug release

Figure S17. HPLC spectra of GEM release *in vitro*

Figure S18. Cellular uptake on RAW 264.7 cells

Table S1. Quantitative data of uptake study corresponding to **Figure 2e**

Figure S19. Cellular uptake on 4T1 cells

Table S2. Quantitative data of uptake study corresponding to **Figure 2f**

Figure S20. Apoptosis assay of 4T1 cells

Figure S21. Flow cytometric analysis of CRT exposure

Figure S22. Gating strategy to determine frequencies of mature DCs

Figure S23. Representative plots of MDSCs elimination *in vitro*

Figure S24. Gating strategy to determine frequencies of *p*-STAT3 positive MDSC *in vitro*

Figure S25. *Ex-vivo* images of excised tumors

Figure S26. Bright field images of 4T1 tumor spheroids

Figure S27. Semi-quantitative analysis of formulations penetrating into the tumor spheroids

Figure S28. Hyaluronic acid (HA) degradation

Figure S29. Representative tumor images

Figure S30. Gating strategy to determine frequencies of mature DCs *in vivo*

Figure S31. Representative plots of mature DCs

Figure S32. Representative plots of TILs in tumor lesions

Figure S33. Representative plots of MDSCs

Figure S34. TUNEL assay of 4T1 tumor

Figure S35. Representative image increased CD8^{+} T cells

Figure S36. MDSC levels in spleen (n=3)

Figure S37. Representative plots of T cells in spleen

Figure S38. Flow cytometric analysis of T cells in primary and abscopal tumor lesions

Figure S39. H&E staining images of major organ sections excised from mice treated with multiple formulations

Table S3. Antibody information

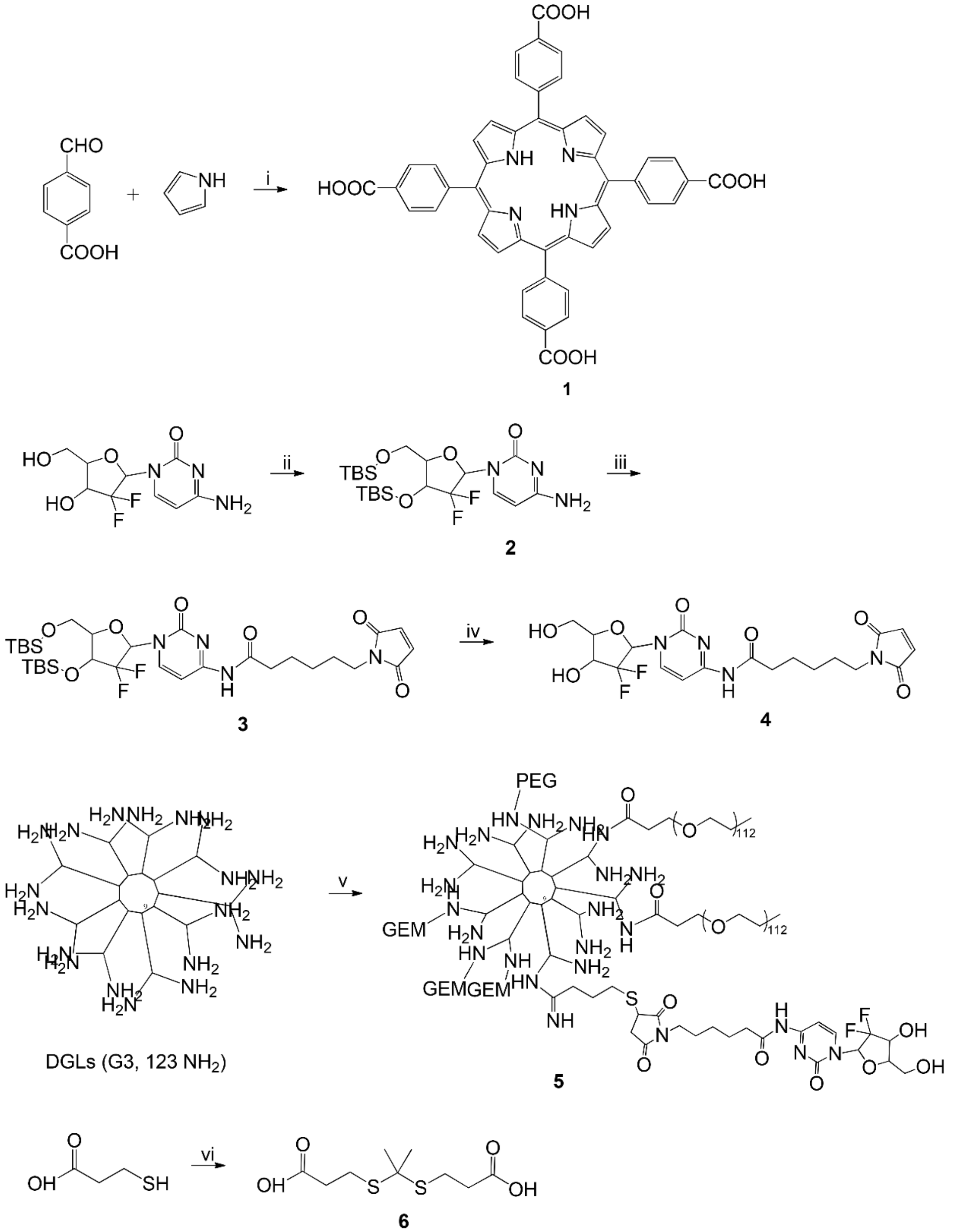

Scheme S1. Synthetic steps and structures of porphyrin, PEG-DGL-GEM polymer and ROS-sensitive linker.

Detailed steps as shown below:

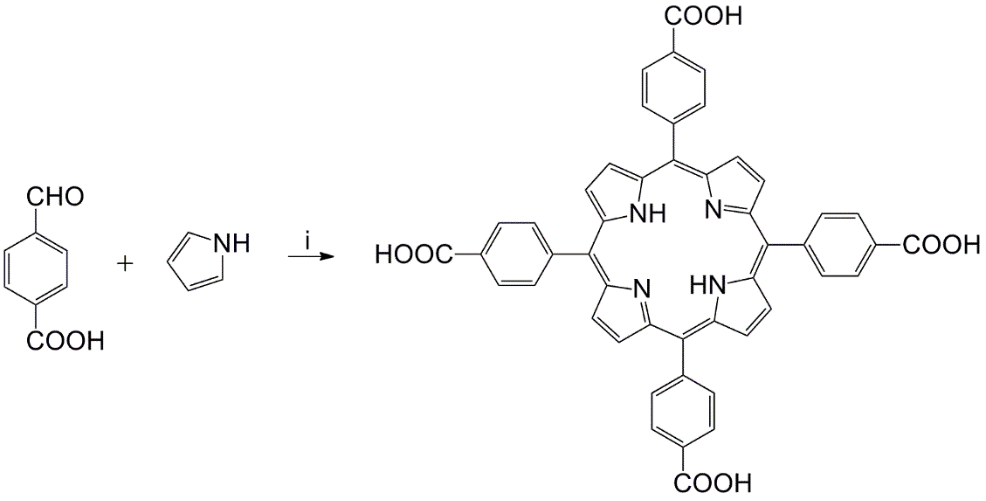

1

The meso-tetra (4-carboxyphenyl) porphine was synthesized according to the following method: pyrrole (10 mmol) was added to a mixture of 4-carboxybenzaldehyde (10 mmol), propionic acid (35 mL). The mixture was heated 2 h at 120 °C, cooled and the solvent was removed under vacuum. The porphyrin was dissolved in 100 mL of 0.2 N NaOH aqueous solution and precipitated with a 1N HCl aqueous solution and then recrystallized in ethanol to give **1**.

^{1}H NMR (400 MHz, DMSO-δ6, δ, ppm): 8.95-8.75 (s, 8H, H-1), 8.43-8.33 (m, 16H, H-2, 3).

MS-ESI Calc. for $C_{48}H_{31}N_4O_8$ [**1**+H]$^+$: 791.21 (100.0%), 792.21 (52.6%), 793.21 (15.8%), 794.22 (2.3%), 792.20 (1.5%), 794.21 (1.1%). Found, 791.21, 192.2, 193.2.

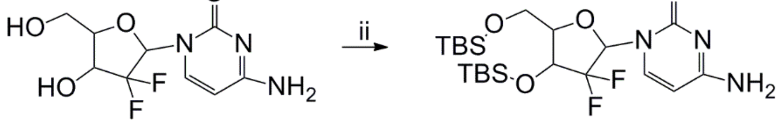

2

This reaction was followed by the reported steps. Gemcitabine HCl (400 mg, 1.33 mmol) and imidazole (1800 mg, 26.44 mmol) was suspended in anhydrous dimethyl sulfoxide

(DMSO, 10 mL) and added with tert-Butylchlorodimethylsilane (3000 mg, 19.90 mmol). The mixture was maintained at r. t. for 2 h. Dichloromethane (DCM, 100 mL) was added to the mixture and washed with NH_4Cl aqueous solution, $NaHCO_3$ solution and saturated NaCl solution (100 mL×3). The organic layer was dried over Na_2SO_4, followed by the removal of solvent. The residual was purified by a column (Methanol: dichloromethane from 0:100 to 10:90, v:v) to afford **2** as a colorless crystal.

R*f* = 0.5 (DCM: methanol=10:1, v:v). s d t q

^{1}H NMR (400 MHz, $CDCl_3$, δ, ppm): 7.70-7.65 (d, 1H, H-21), 6.38-6.28 (m, 1H, H-20), 5.75-5.68 (d, 1H, H-22), 4.35-4.25 (q, 1H, H-18), 4.02-3.93 (d, 1H, H-16), 3.91-3.84 (d, 1H, H-17), 3.80-3.72 (d, 1H, H-19), 0.95-0.86 (d, 18H, H-1-9), 0.14-0.08 (s, 12H, H-10-15).

MS-ESI Calc. for $C_{21}H_{40}F_2N_3O_4Si_2$ [**2**+H]$^+$ 492.24 (100.0%), 493.25 (23.3%), 493.24 (11.3%), 494.24 (7.1%), 494.25 (6.0%), 495.24 (2.0%). Found, 492.31

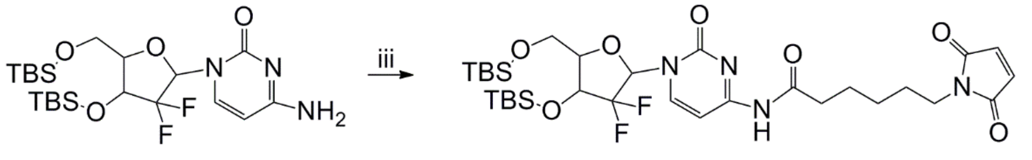

2 **3**

The protocol was followed by the previous literature. Compound **2** (100 mg, 0.203 mmol), 6-maleimidohexanoic acid (85.9 mg, 0.407 mmol), 2-(7-Azabenzotriazol-1-yl)-*N,N,N',N'*-tetramethyluronium hexafluorophosphate (HATU, 185.4 mg, 0.488 mmol) and *N,N*-Diisopropylethylamine (DIPEA, 62 mg, 0.488 mmol) were suspended in anhydrous dimethyl formamide (DMF, 10 mL) under Ar/r. t. for 24 h ,and then extracted with ethyl acetate (20 mL×3). The organic layer was dried over Na_2SO_4, followed by the removal of solvent. The residual was purified by a column (petroleum: ethyl acetate

from 80:20 to 60:40, v:v) to afford **3** as a colorless crystal.

R*f* = 0.42 (petroleum: ethyl acetate =1:1, v:v).

^{1}H NMR (400 MHz, $CDCl_3$, δ, ppm): 8.15-8.08 (d, 1H, H-22), 7.50-7.43 (d, 1H, H-21), 6.72-6.67 (s, 2H, H-33, 34), 6.36-6.30 (d, 1H, H-20), 4.40-4.25 (q, 1H, H-18), 4.10-3.95 (m, 2H, H-16, 19), 3.85-3.78 (d, 1H, H-17), 3.55-3.45 (t, 2H, H-31, 32), 2.60-2.50 (t, 2H, H-23, 24), 1.75-1.58 (m, 4H, H-25, 26, 29, 30), 1.42-1.30 (m, 2H, H-27, 28), 0.95-0.86 (d, 18H, H-1-9), 0.21-0.08 (s, 12H, H-10-15).

MS-ESI Calc. for $C_{31}H_{51}F_2N_4O_7Si_2$ [**3**+H]$^+$ 685.32 (100.0%), 686.32 (46.0%), 687.32 (12.5%), 687.33 (5.7%), 688.32 (3.6%), 688.33 (1.1%). Found, 685.2, 686.2, 687.2, 688.2

$C_{31}H_{50}F_2NaN_4O_7Si_2$ [**3**+Na]$^+$ 707.32 (100.0%), 708.32 (46.0%), 709.32 (12.5%), 709.33 (5.7%), 710.32 (3.6%), 710.33 (1.1%). Found, 707.2, 708.2, 709.2

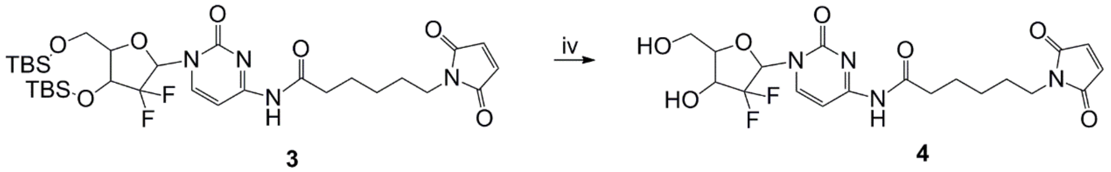

Compound **3** (100 mg, 0.146 mmol) was dissolved in anhydrous tetrahydrofuran (5 mL), 50 μL Acetic acid and 500 μL tetrabutylammonium fluoride (1 mmol/mL) was added, and stirred at ice-water bath for 5 h under Ar. After removal of solvent, the residual was purified by a column (Methanol: dichloromethane at 9:91, v:v) to afford **4** as a colorless crystal.

R*f* = 0.3 (DCM: methanol =20:1, v:v).

For **4**: ^{1}H NMR (400 MHz, DMSO-δ6, δ, ppm): 8.27-8.20 (d, 1H, H-7), 7.31-7.25 (d, 1H, H-6), 7.05-6.95 (s, 2H, H-18, 19), 6.36-6.30 (d, 1H, H-5), 6.21-6.15 (m, 1H, H-16),

5.37-5.28 (t, 1H, H-17), 4.25-4.10 (m, 1H, H-3), 3.92-3.85 (m, 1H, H-1), 3.85-3.75 (m, 1H, H-2), 3.70-3.60 (m, 1H, H-4), 2.45-2.35 (m, 2H, H-8, 9), 1.60-1.15 (m, 6H, H-10~15).

MS-ESI Calc. for $C_{19}H_{23}F_2N_4O_7$ [**4**+H]$^+$ 457.15 (100.0%), 458.15 (21.1%), 459.15 (3.8%), 458.14 (1.5%). Found, 457.0, 458.0

$C_{19}H_{22}F_2NaN_4O_7$ [**4**+Na]$^+$ 479.15 (100.0%), 480.15 (21.1%), 481.15 (3.8%), 480.14 (1.5%). Found, 476.2, 479.0

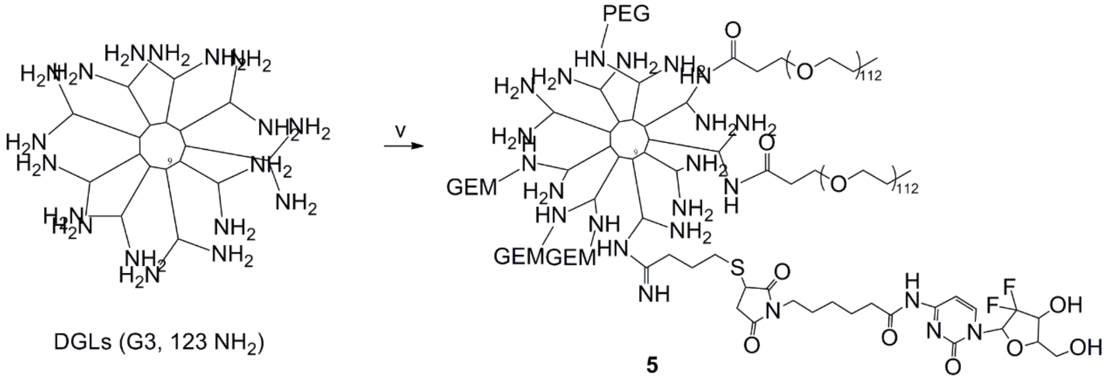

Dendri-graft-*L*-lysine (G3, 30 mg, 0.00136 mmol), 2-iminothiolane hydrochloride (3 mg, 0.0218 mmol), triethylamine (4.5 mg, 0.0445 mmol), compound **4** (9.3mg, 0.0204 mmol) and CH_3O-PEG-NHS (30 mg, 0.006 mmol) were dissolved in anhydrous DMF (5 mL) under Ar/r. t. for 24 h. the mixture was dialysis against H_2O for 96 h and freeze-dried to give **5** as a white powder.

For **5**: ^{1}H NMR (400 MHz, DMSO-δ6, δ, ppm): 7.35-7.29 (m, 14H, H-1), 6.25-6.18 (m, 14H, H-2), 3.54-3.45 (m, H-protons of PEG), 2.15-0.8 (m, H-protons of DGLs)

For **5**: the calculated molecule weight of compound **5** measured by GPC analysis was 56940 (Mw).

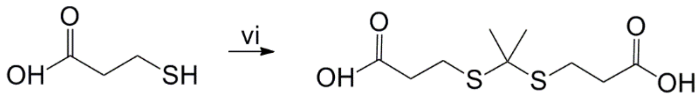

6

3-mercaptopropionic acid (3 g, 28.3 mmol) and acetone (3.35 g, 56.7 mmol) were stirred under a dry hydrogen chloride condition at room temperature for 6 h. After the reaction, the flask was stoppered and chilled in an ice-salt mixture until crystallization was complete. The crystals were filtered, washed with hexane and cold water, and get **6** as a white crystal.

^{1}H NMR (400 MHz, Methanol-δ4, δ, ppm): 2.88-2.82 (t, 4H, H-4, 5), 2.62-2.56 (t, 4H, H-6, 7), 1.60-1.57 (s, 6H, H-1~3).

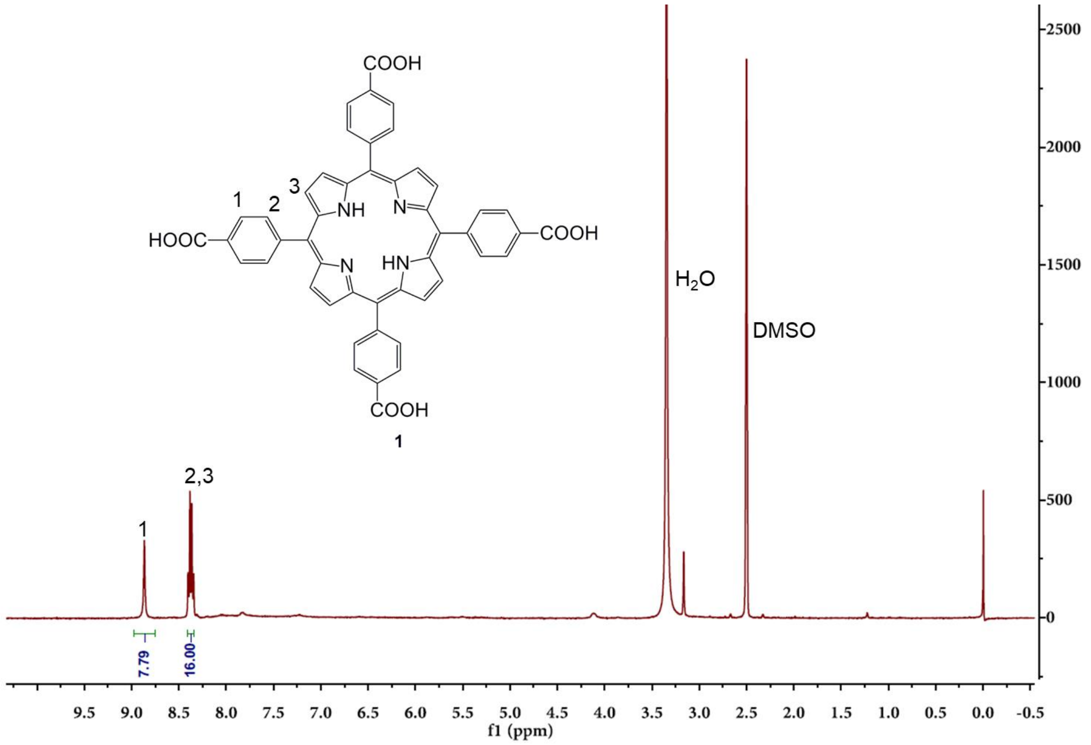

Figure S1. The ^{1}H NMR spectrum of **1** in DMSO-δ6.

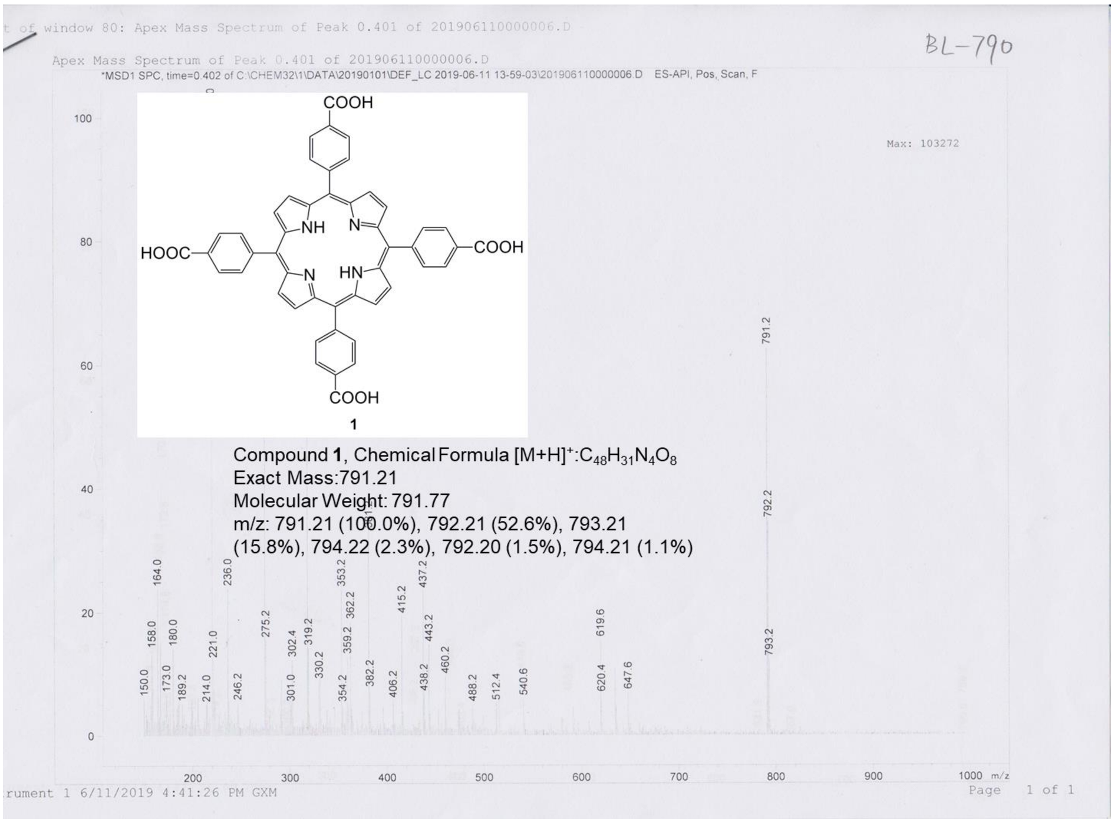

Figure S2. The MS-ESI spectrum of **1**.

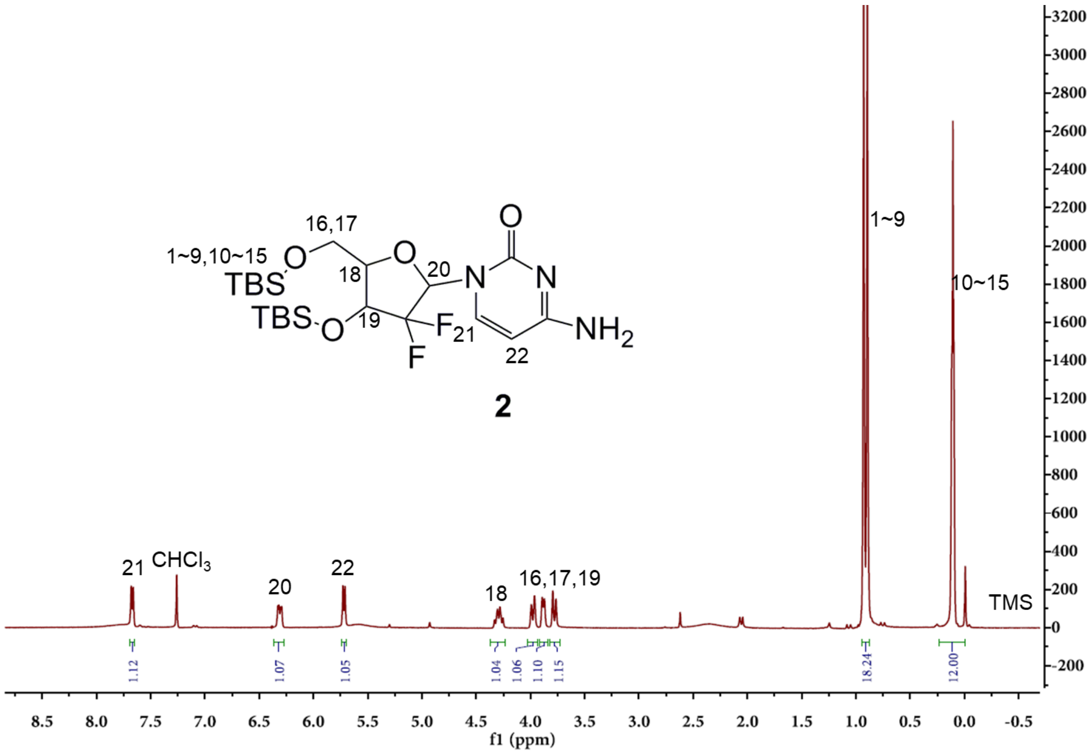

Figure S3. The ^{1}H NMR spectrum of **2** in $CDCl_3$.

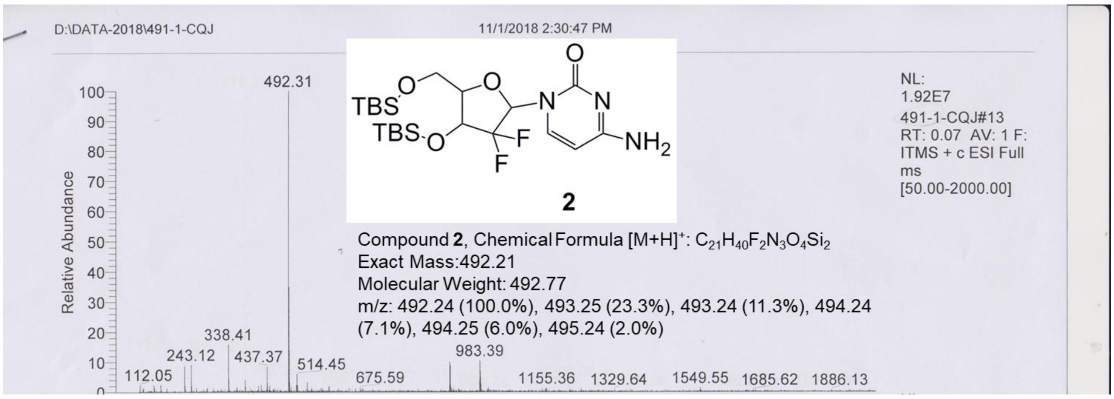

Figure S4. The MS-ESI spectrum of **2**.

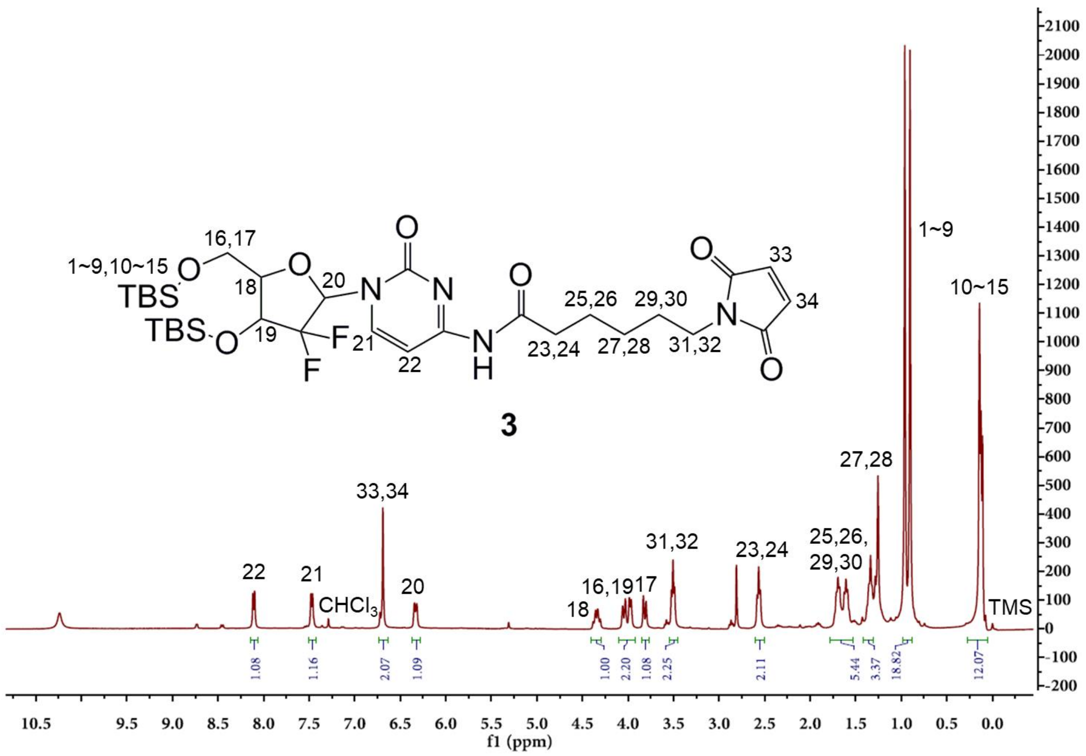

Figure S5. The ^{1}H NMR spectrum of **3** in $CDCl_3$.

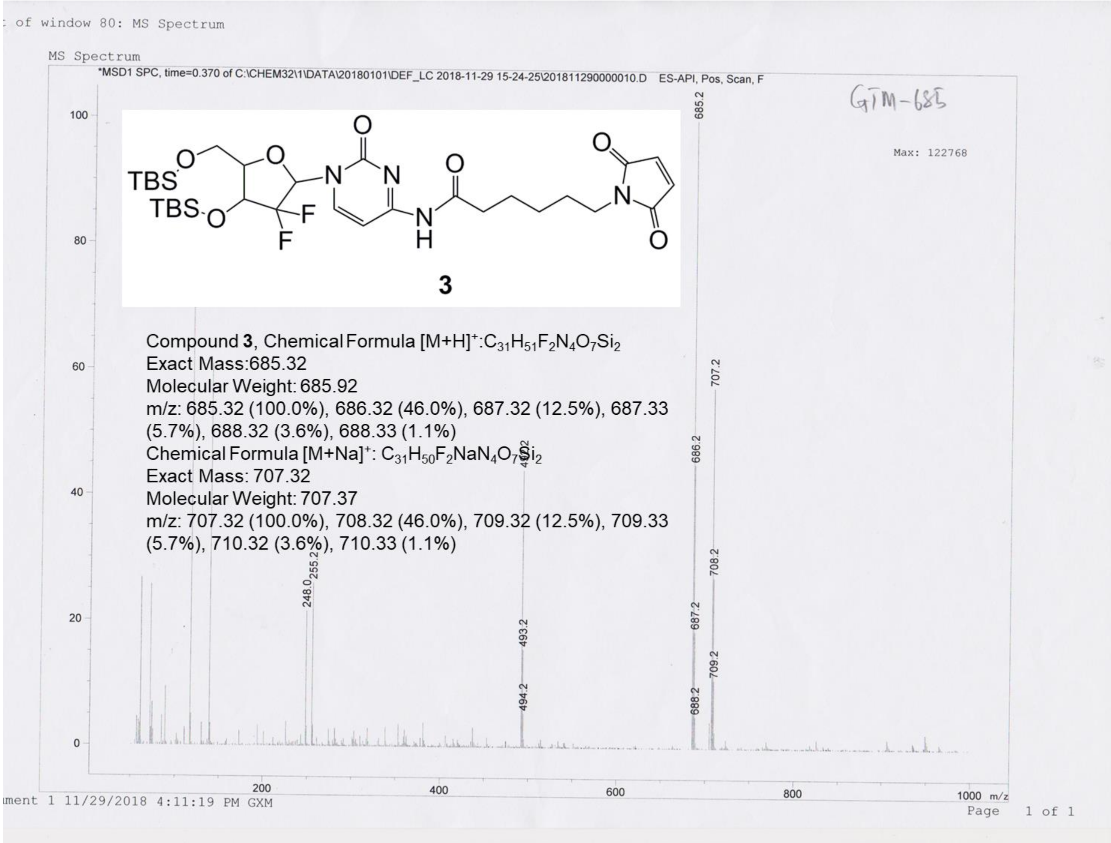

Figure S6. The MS-ESI spectrum of **3**.

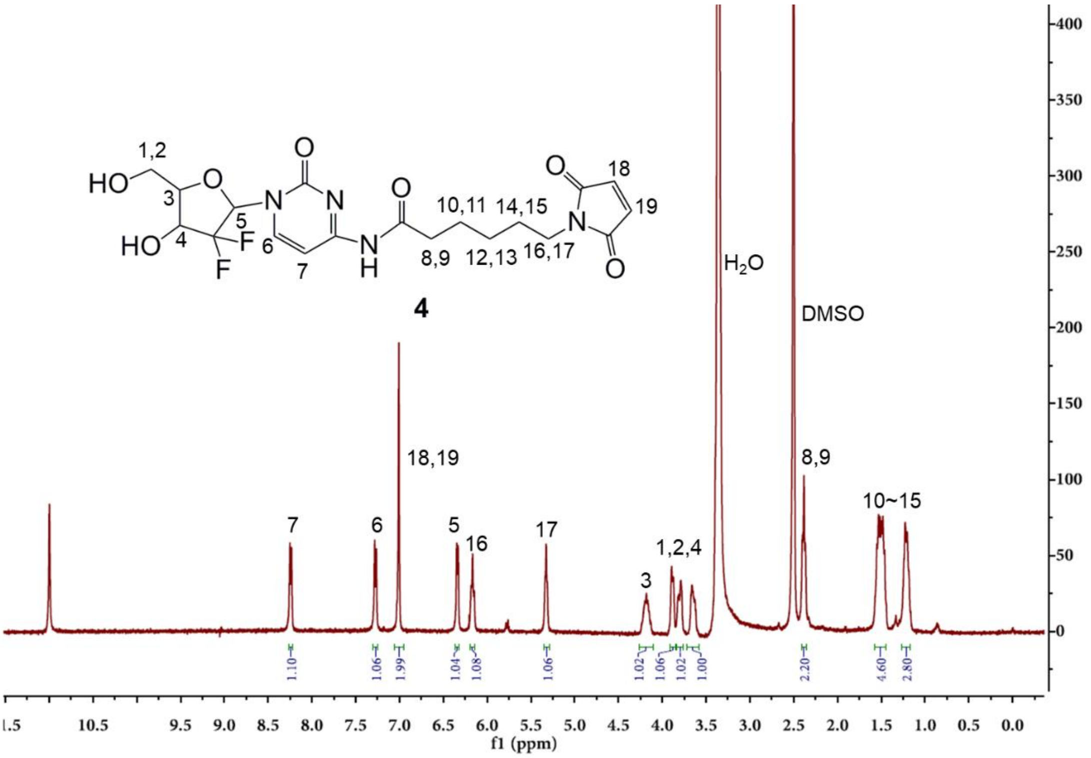

Figure S7. The ^{1}H NMR spectrum of **4** in DMSO-δ6.

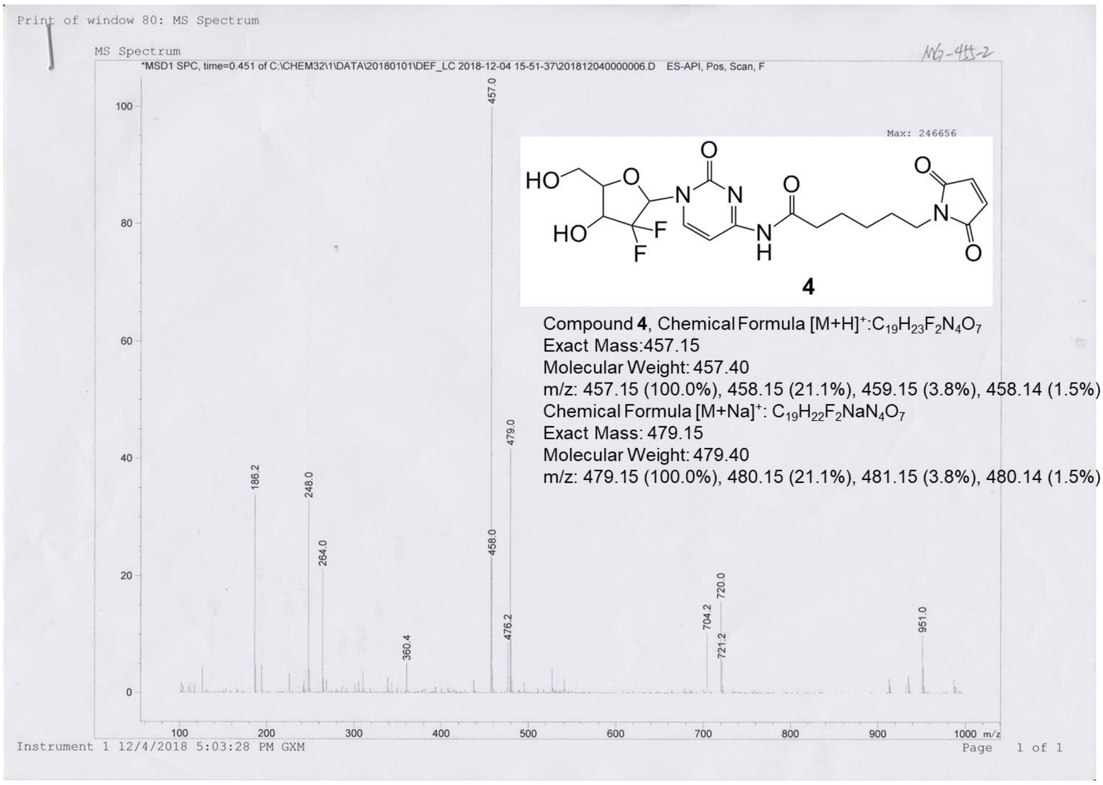

Figure S8. The MS-ESI spectrum of **4**.

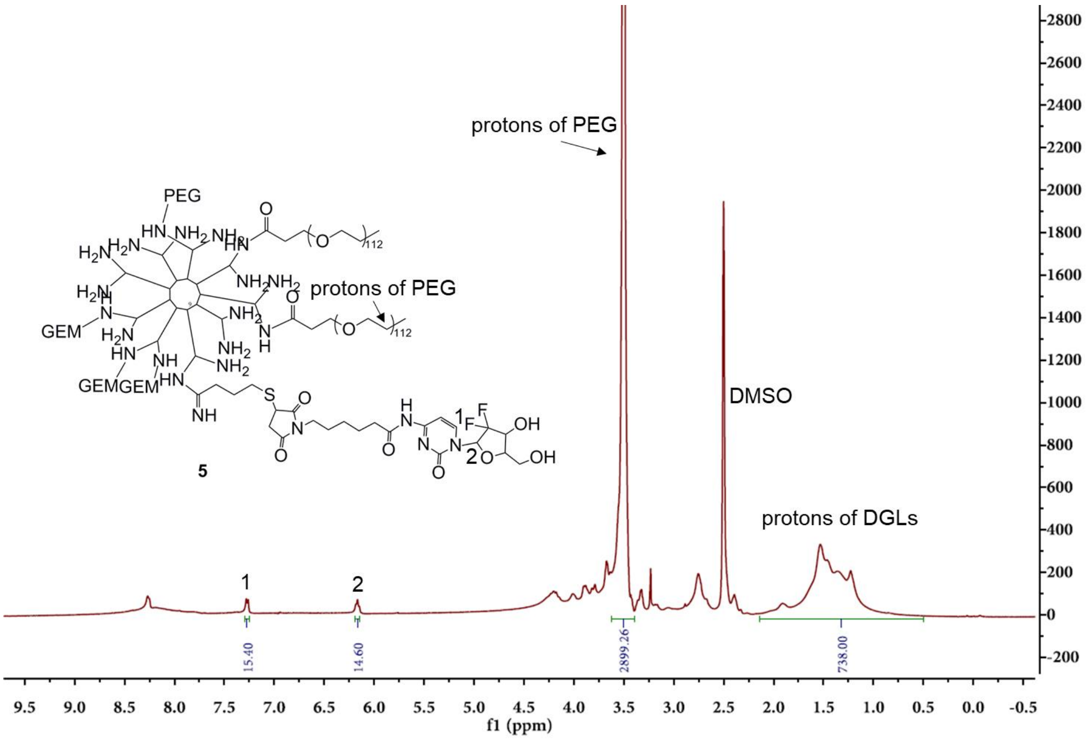

Figure S9. The ^{1}H NMR spectrum of **5** in DMSO-δ6.

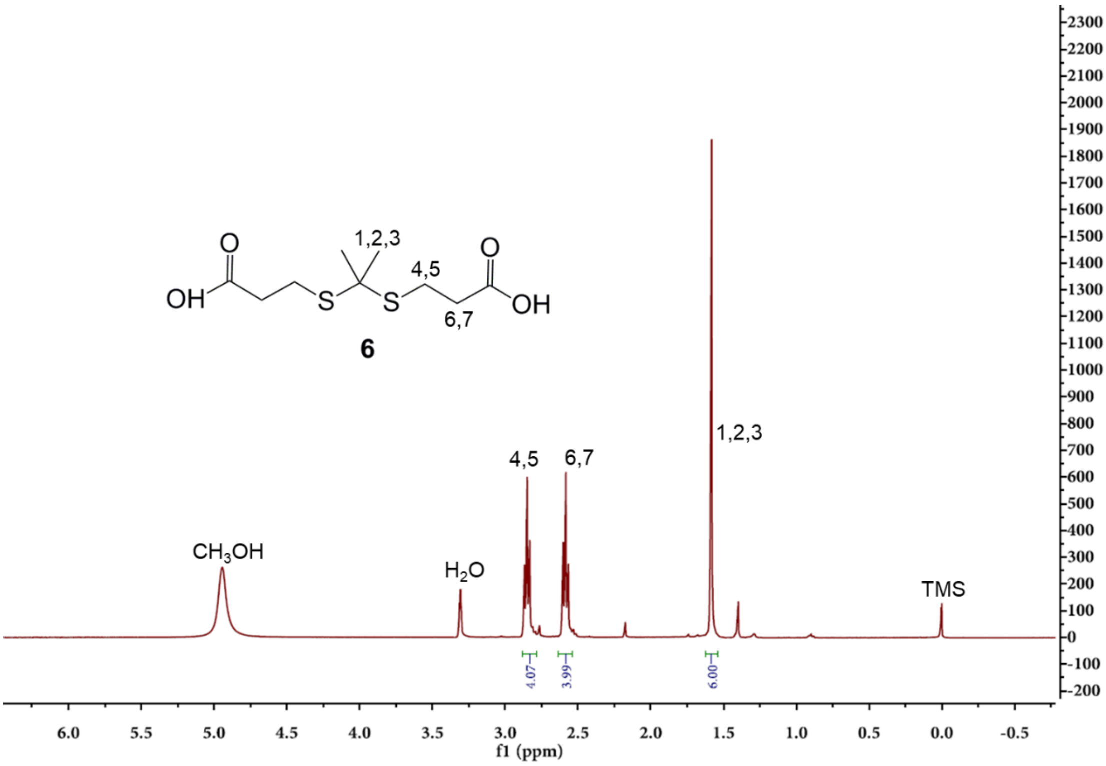

Figure S10. The ^{1}H NMR spectrum of **6** in Methanol-δ4.

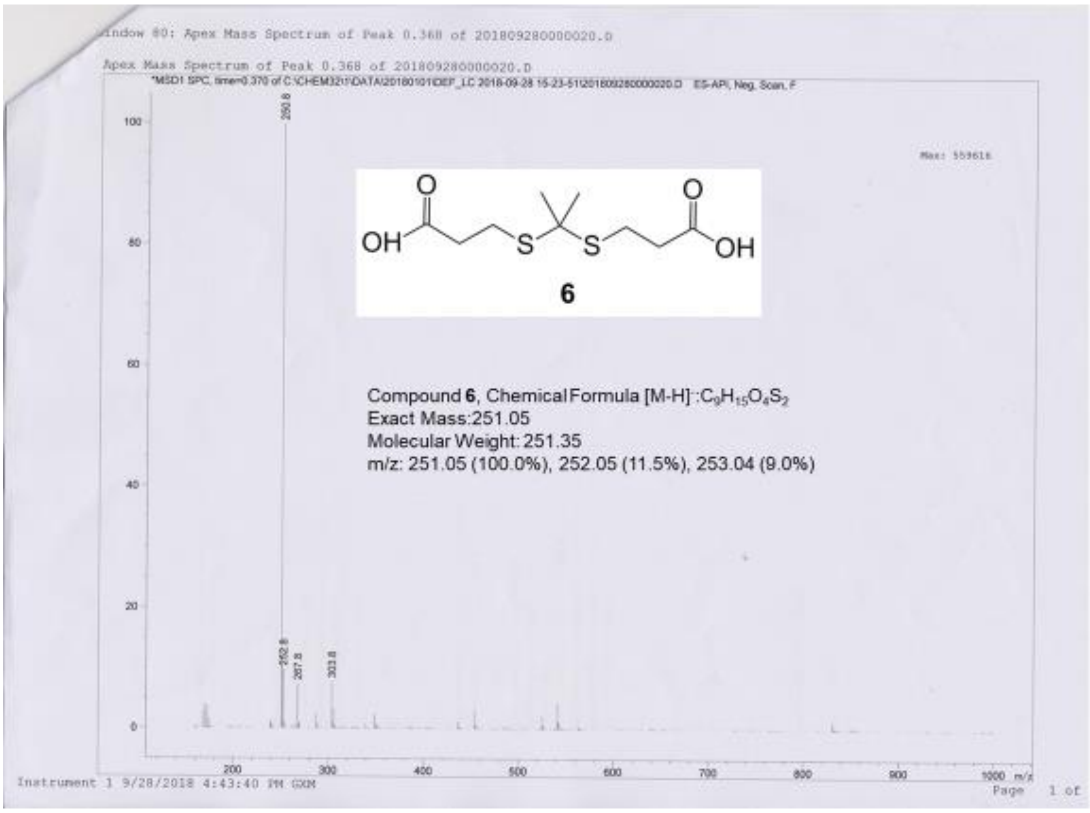

Figure S11. The MS-ESI spectrum of **6**.

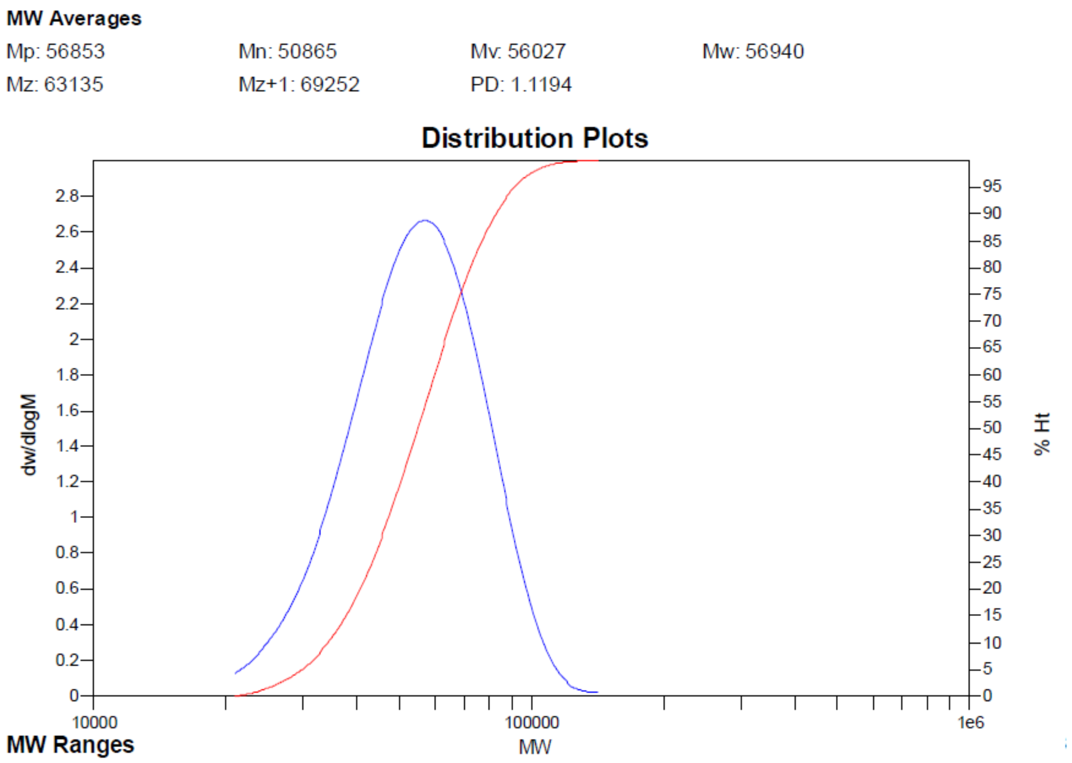

Figure S12. GPC result and calculated molecule weight of **5**.

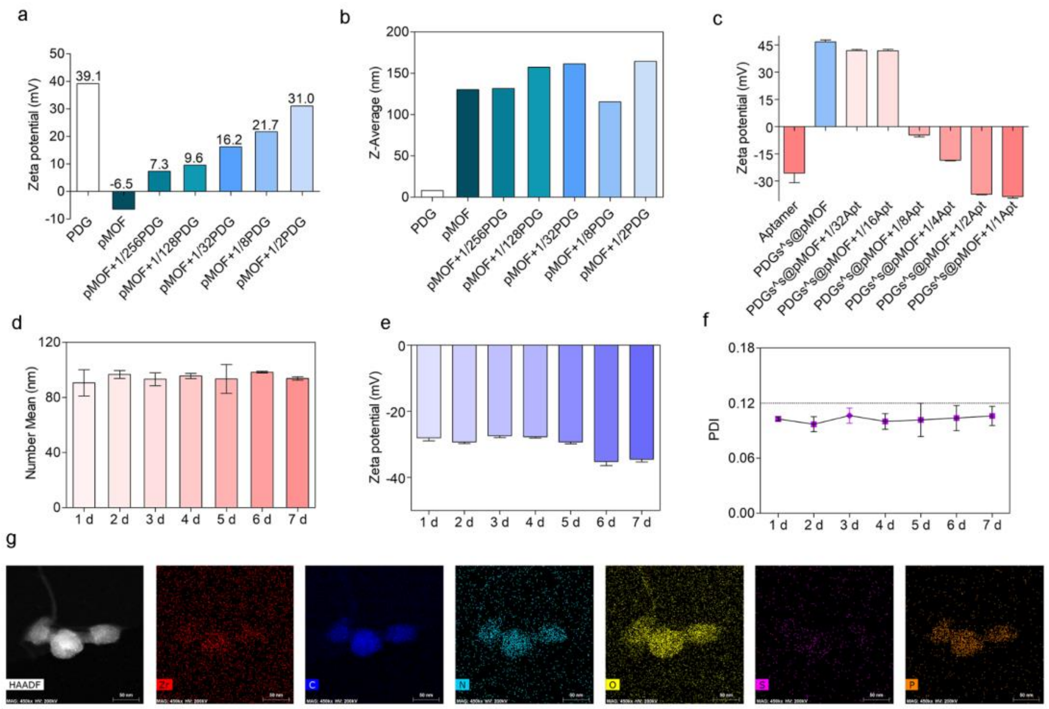

Figure S13. **a**. Variation of zeta potential value during concentration-dependent increased PDG binding on the surface of pMOF nanoparticles, and the mass ratio of PDG to pMOF was increased from 1/256 to 1/2. **b**. Changes of DLS profiles during

concentration-dependent increased PDG binding on the surface of pMOF nanoparticles, and the mass ratio of PDG to pMOF was increased from 1/256 to 1/2. **c**. Variation of zeta potential value during concentration-dependent increased aptamer binding on the surface of PDGs^s@pMOF nanoparticles, and the mass ratio of aptamer to PDG was increased from 1/256 to 1/2. **d, e, f**. the changes of hydrous diameters, zeta potential, and PDI value of Apt/PDGs^s@pMOF during incubation with DMEM medium (containing 10% FBS) for 7 day. **g**. HAADF image of non-cross-linked Apt/PDG@pMOF and the distribution for the elemental mapping of Zr, C, N, O, S, P. Scale bars: 30 nm.

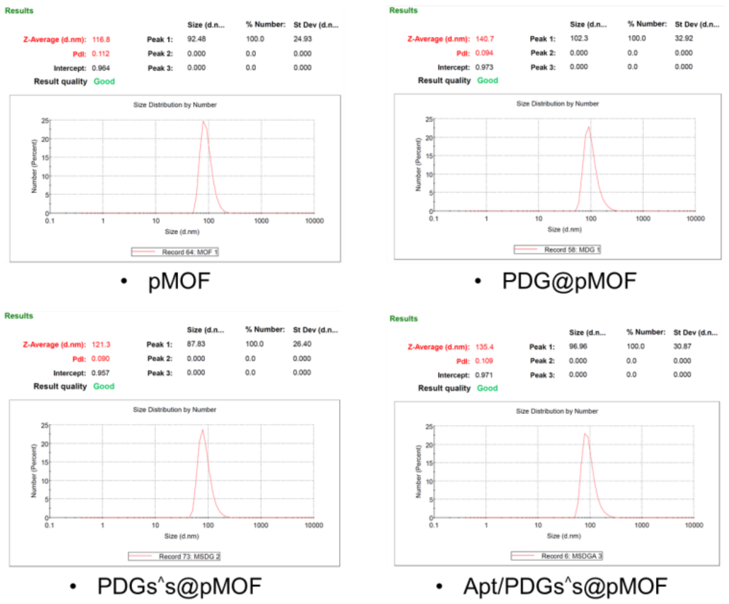

Figure S14. DLS histogram data in the lay-by-lay process of Apt/PDGs^s@pMOF preparation.

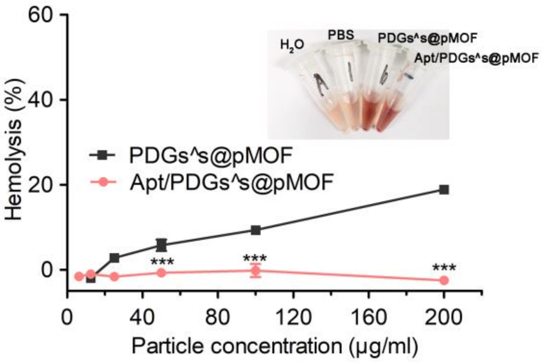

Figure S15. Percentage of hemolysis of RBCs in the presence of two kinds of nanoparticles at different concentrations. The inset shows the photograph results of the highest concentration. Significance is defined as *** P < 0.001.

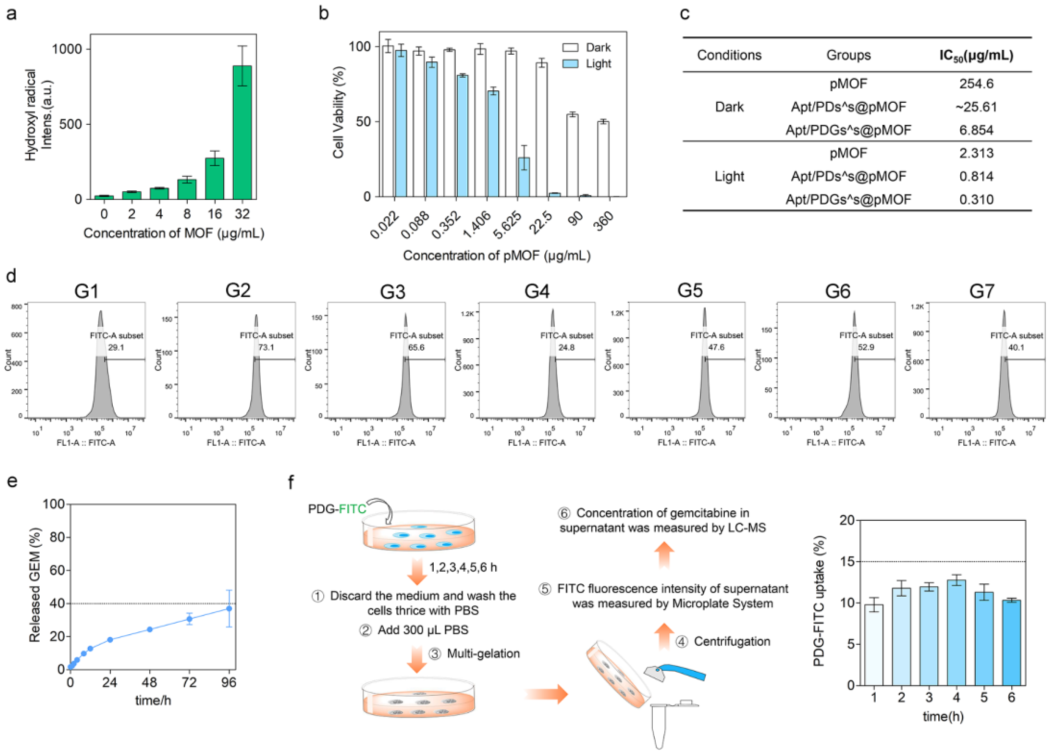

Figure S16. a. Hydroxyl radical generation upon PDT treatment with increasing concentration of pMOF. Data are presented as means ± SD (n=4). **b**. CCK-8 assay of 4T1 cells after 48-h treatment with various concentrations of pMOF with or without irradiation for 5 min under 660 nm laser. Data are presented as means ± SD (n=4). **c**. Summary table of IC50 values. **d**. Flow cytometric analysis of ROS generation. **e**.

Sustained GEM release profile of PDG polymer in PBS 7.4. Data are presented as means ± SD (n=3). **f.** Experimental steps of intracellular released-drug detection and time-dependent variation of intracellular uptake of PDG-FITC. Data are presented as means ± SD (n=3). G1: Control + L (laser irradiation), G2: Apt/PDGs^s@pMOF + L, G3: cApt/PDGs^s@pMOF + L, G4: Apt/PDG@pMOF + L, G5: Apt/PDGs^s@pMOF (P-L: pre-irradiated) + L, G6: Apt/PDGs^s@pMOF (+NAC) + L, G7: Apt/PDGs^s@pMOF + D (dark).

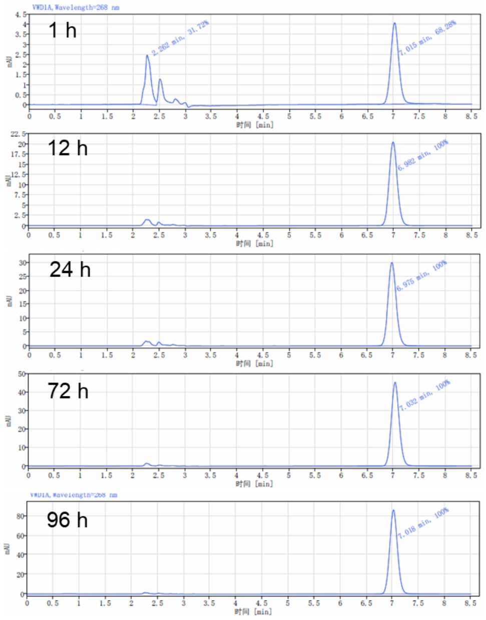

Figure S17. The HPLC spectra of supernatants of dissolution medium in which PDG polymers was incubated with PBS 7.4 at different times.

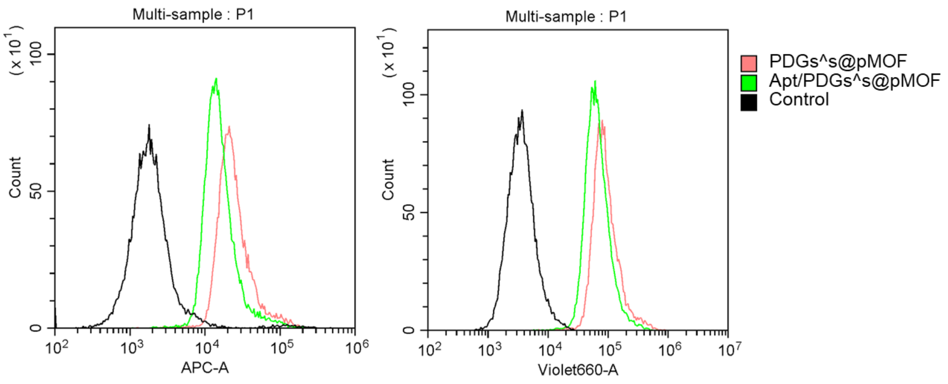

Figure S18. Cellular uptake of Apt/PDGs^s@pMOF and PDGs^s@pMOF on RAW 264.7 cells by flow cytometry analysis, based on the fluorescence signal of Cy5.5-labeled PDG and pMOF, respectively.

Groups	Mean FITC	Mean pMOF	FITC/pMOF
Apt/PDGs^s@pMOF	6215.9	10104	0.62
cApt/PDGs^s@pMOF	5378.8	7627	0.70
Apt/PDGs^s@pMOF(P-L)	4239.1	5149.6	0.82
Apt/PDG@pMOF	4136.4	4019.2	1.03

Table S1. The Quantitative data of uptake study, which was corresponding to **Figure 2e** (Gain value: FSC (75), SSC (174), FITC (15), Violet660 (pMOF, 155)).

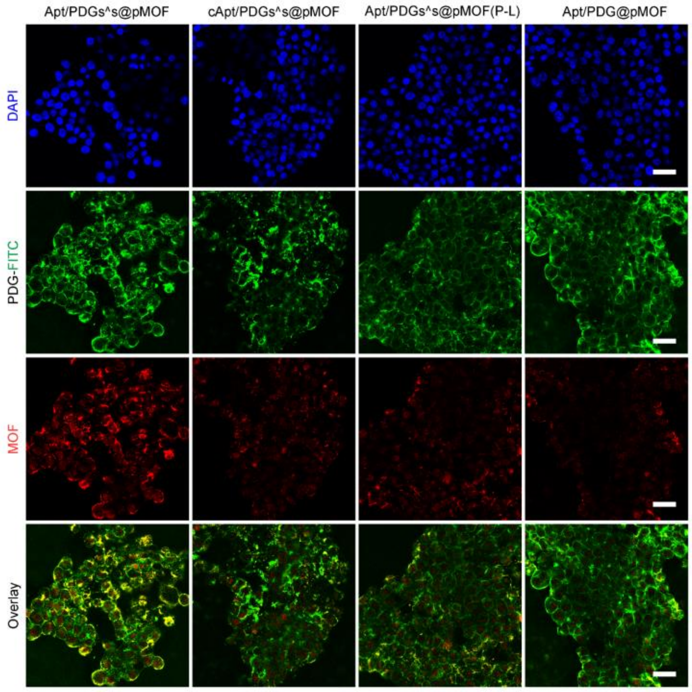

Figure S19. Cellular uptake of different formulations on 4T1 cells by detecting the fluorescence signal of FITC-labeled PDG and pMOF under CLSM ((P-L): pre-irradiated). Scale bars: 40 μm.

Groups	Mean FITC	Mean TRITC	Mean pMOF	FITC/pMOF	TRITC/pMOF
Apt/PDGs^s@pMOF	2285.1	2236.7	6979.8	0.33	0.32
+Colchicine	2211.5	2556.3	5732.4	0.33	0.34
+Filipin	2570.1	2589.6	7726.8	0.39	0.45
4°C	1250.8	1682.4	3161.2	0.40	0.53
+PAsO	1119.0	3342.4	2819.7	0.40	1.19

Table S2. The Quantitative data of uptake study, which was corresponding to **Figure 2f** (Gain value: FSC (75), SSC (174), FITC (1), ECD (TRITC, 197), Violet660 (pMOF, 100)).

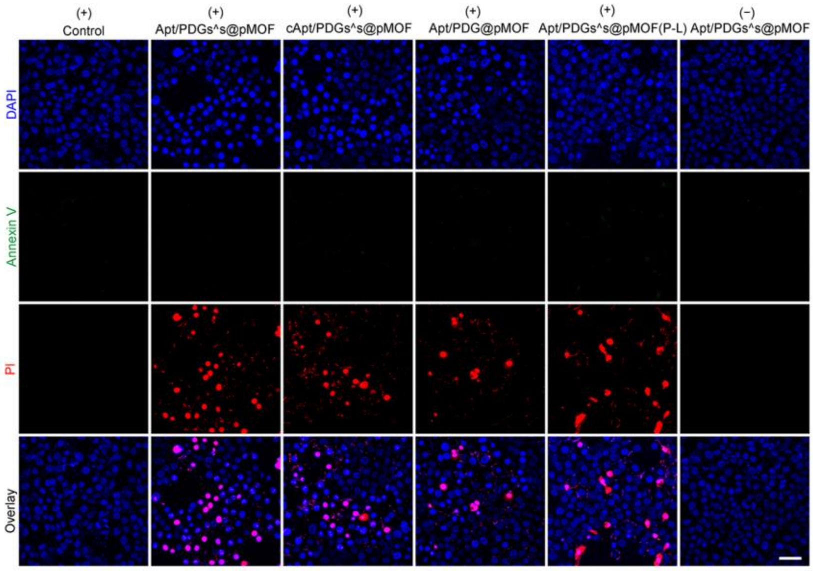

Figure S20. Apoptosis assay of 4T1 cells by CLSM ((+): laser irradiation, (-): dark, (P-L): pre-irradiated). As shown in this figure, there was no annexin V detected while the cytomembrane of PDT-treated cells were broken (positve PI staining), indicating that the PDT-induced cell death was independent of classical apoptosis. Scale bars: 50 μm.

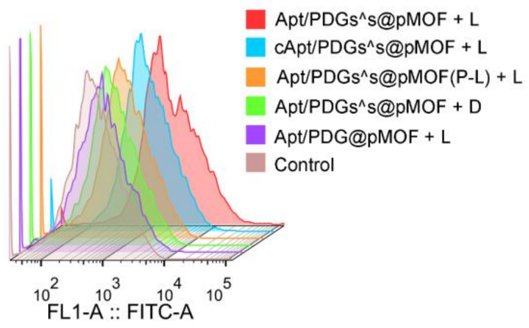

Figure S21. Flow cytometric analysis of CRT exposure (+ L: laser irradiation, (P-L): pre-irradiated, + D: dark).

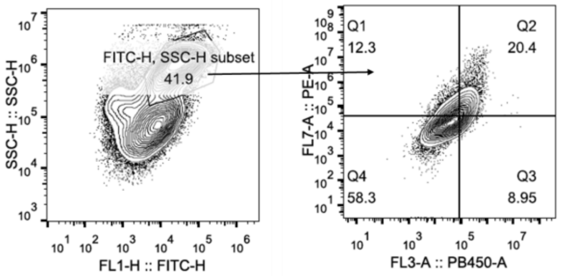

Figure S22. Gating strategy to determine frequencies of mature DCs *in vitro*.

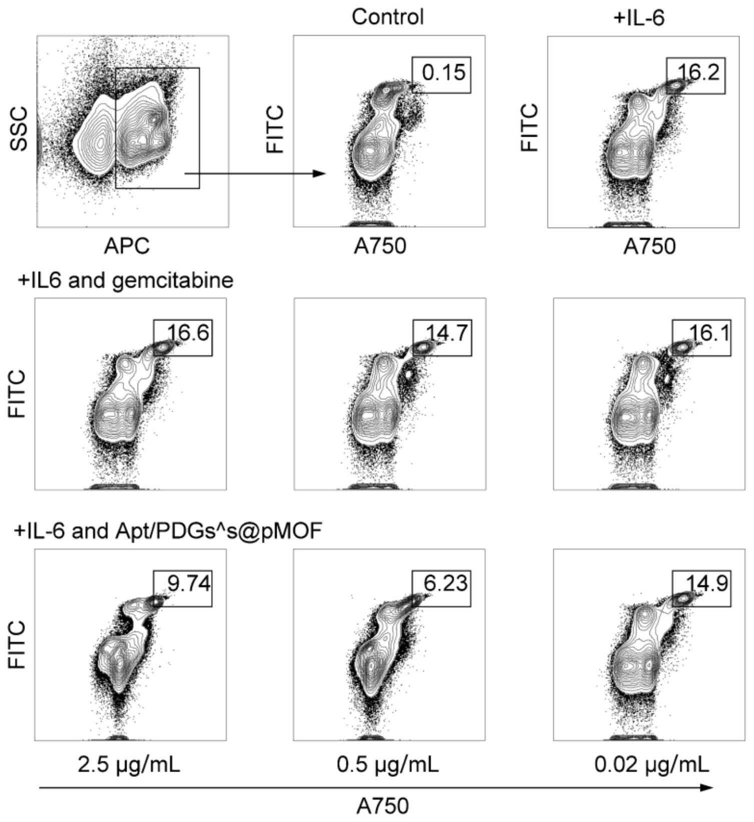

Figure S23. Representative plots of MDSCs elimination *in vitro*.

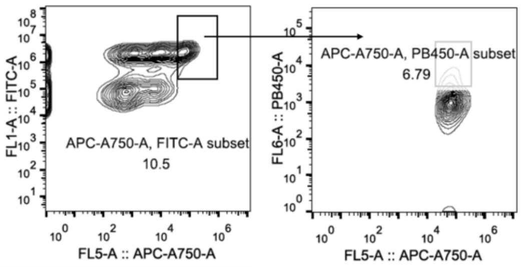

Figure S24. Gating strategy to determine frequencies of *p*-STAT3 positive MDSC *in vitro*.

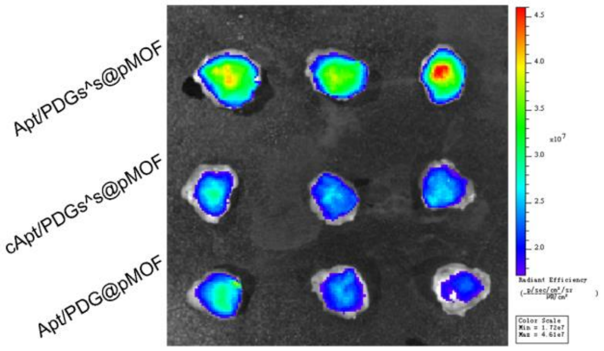

Figure S25. *Ex-vivo* images of excised tumors isolated from tumor-bearing mice by IVIS at 48 h post the injection.

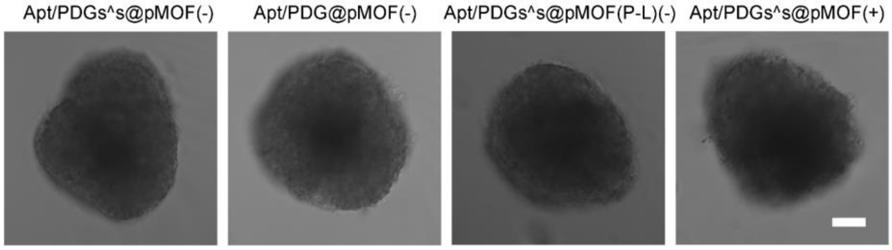

Figure S26. Bright field images of 4T1 tumor spheroids ((-): dark, (P-L): pre-irradiated, (+): laser irradiation). Scale bars: 75 μm.

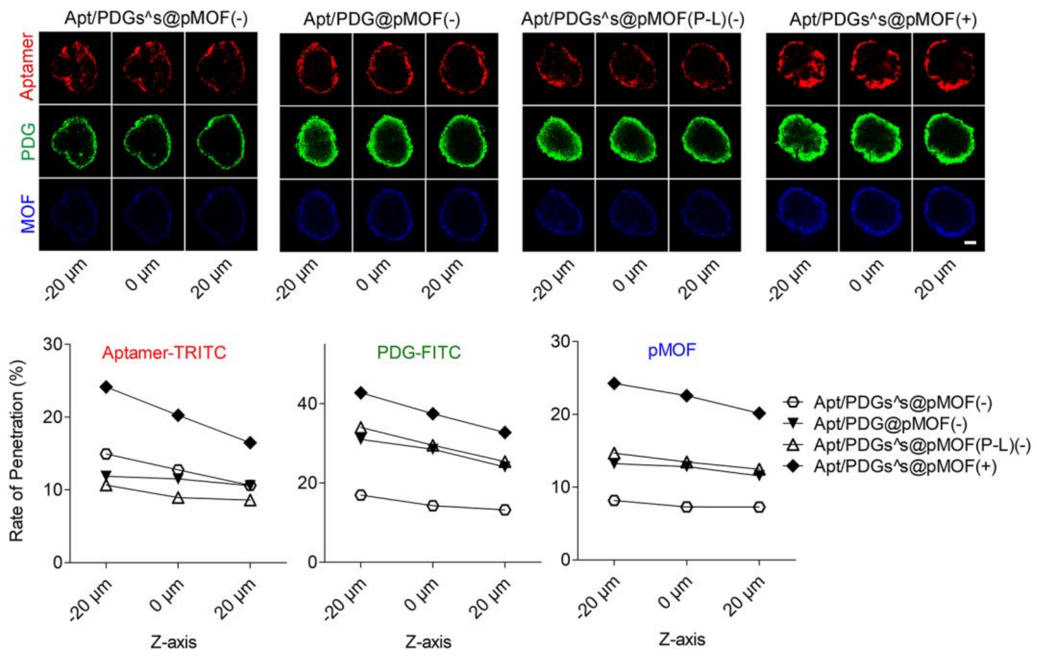

Figure S27. Semi-quantitative analysis of formulations penetrating into the tumor spheroid *via* ImageJ by measuring the percentage of fluorescent area in tumor sphere area ((-): dark, (+): laser irradiation, (P-L): pre-irradiated). Scale bars: 75 μm.

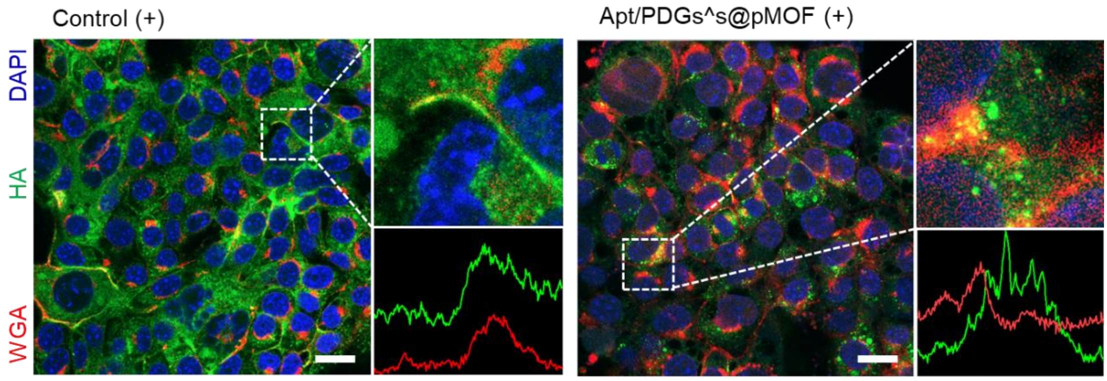

Figure S28. Hyaluronic acid (HA) degradation after treatment with PDT ((+): laser irradiation). Scale bars: 20 μm.

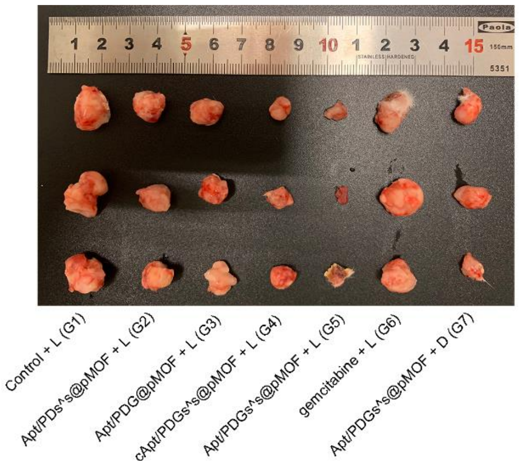

Figure S29. Representative tumor images at day 18 post-implantation of primary side (+L: laser irradiation, +D: dark).

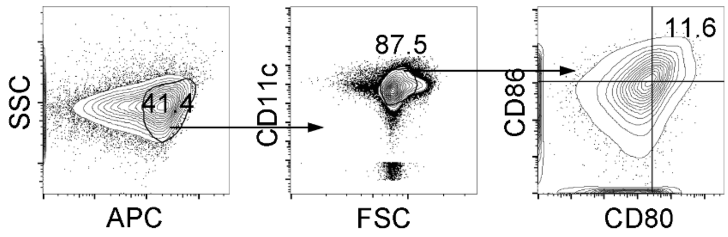

Figure S30. Gating strategy to determine frequencies of mature DCs *in vivo*.

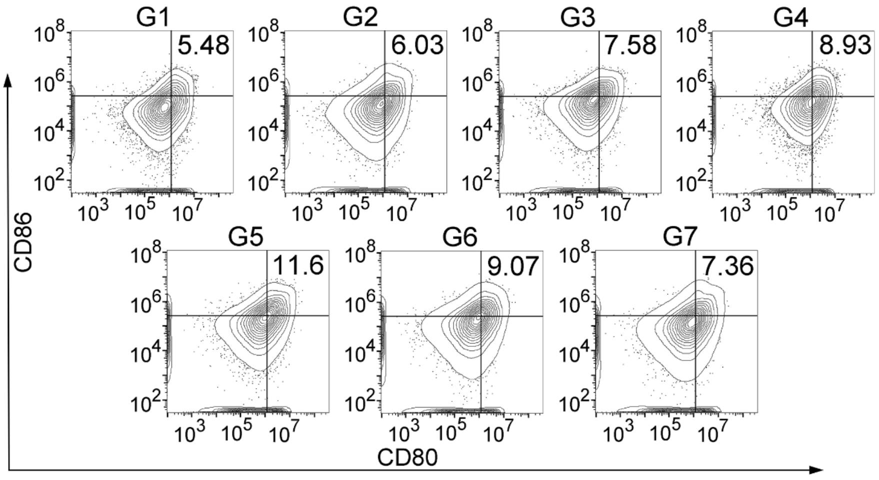

Figure S31. Representative plots of mature DCs in tumor draining lymph nodes in different groups. G1: Control + L (laser irradiation), G2: Apt/PDs^s@pMOF + L, G3: Apt/PDG@pMOF + L, G4: cApt/PDGs^s@pMOF + L, G5: Apt/PDGs^s@pMOF + L,

G6: GEM + L, G7: Apt/PDGs^s@pMOF + D (dark).

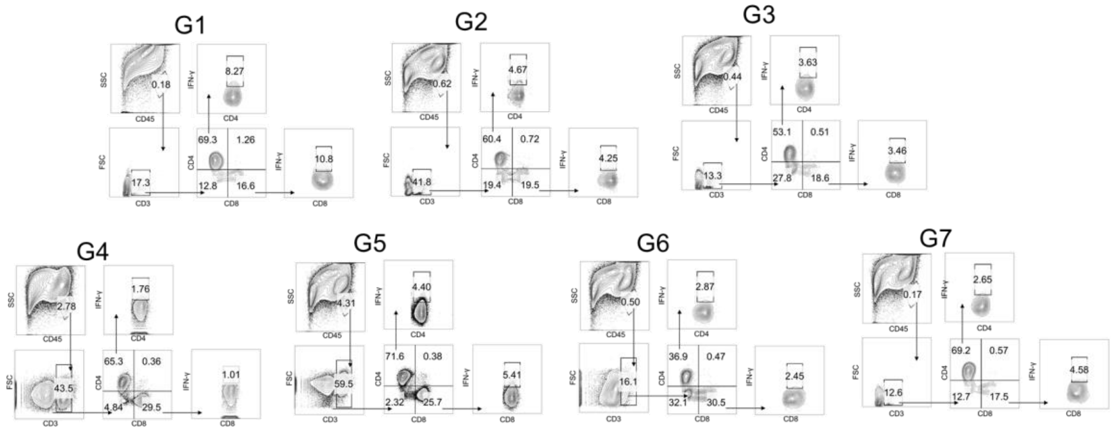

Figure S32. Representative plots of TILs in tumor lesions. G1: Control + L (laser irradiation), G2: Apt/PDs^s@pMOF + L, G3: Apt/PDG@pMOF + L, G4: cApt/PDGs^s@pMOF + L, G5: Apt/PDGs^s@pMOF + L, G6: GEM + L, G7: Apt/PDGs^s@pMOF + D (dark).

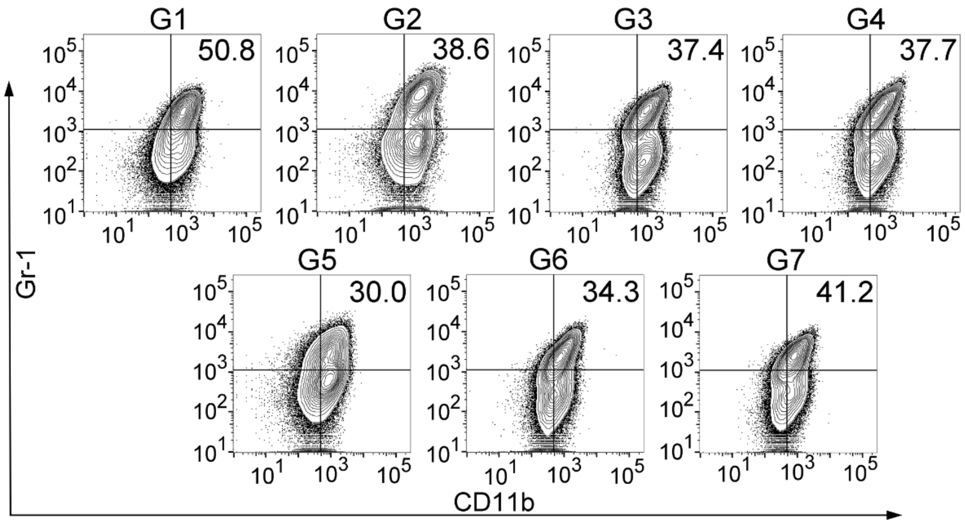

Figure S33. Representative plots of MDSCs in tumor lesions in different groups. G1: Control + L (laser irradiation), G2: Apt/PDs^s@pMOF + L, G3: Apt/PDG@pMOF + L, G4: cApt/PDGs^s@pMOF + L, G5: Apt/PDGs^s@pMOF + L, G6: GEM + L, G7: Apt/PDGs^s@pMOF + D (dark).

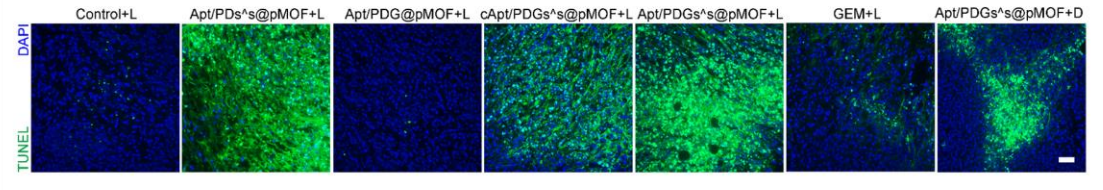

Figure S34. TUNEL assay of 4T1 tumor xenografts excised from mice models (+L: laser irradiation, +D: dark). Scale bars: 50 μm.

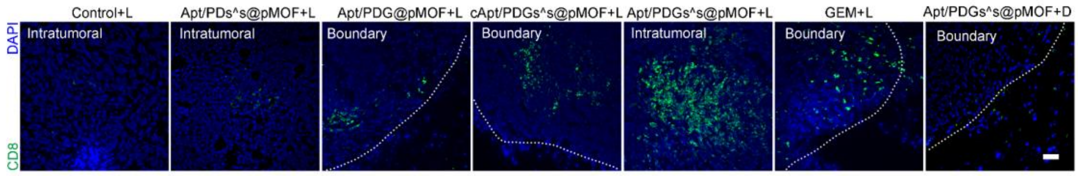

Figure S35. Representative image increased $CD8^+$ T cells tumor-infiltration (+L: laser irradiation, +D: dark). Scale bar: 50 μm.

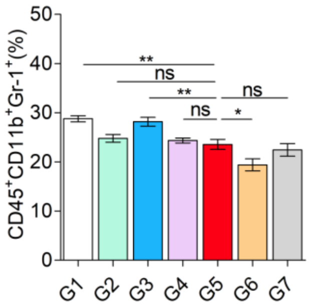

Figure S36. MDSC levels in spleen (n=3). G1: Control + L (laser irradiation), G2: Apt/PDs^s@pMOF + L, G3: Apt/PDG@pMOF + L, G4: cApt/PDGs^s@pMOF + L, G5: Apt/PDGs^s@pMOF + L, G6: GEM + L, G7: Apt/PDGs^s@pMOF + D (dark). Significance is defined as ns, no significance, ⋆ $P < 0.05$, ⋆⋆ $P < 0.01$.

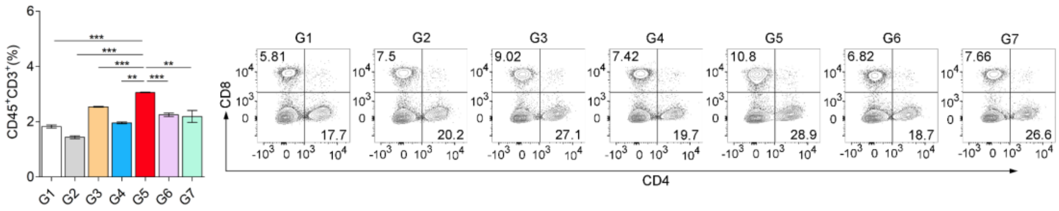

Figure S37. $CD3^+$ T cells levels and representative plots of $CD8^+$ T cell and $CD4^+$ T cells in spleen of tumor-bearing mice after various treatments, analyzed by flow

cytometry (n=3). G1: Control + L (laser irradiation), G2: Apt/PDs^s@pMOF + L, G3: Apt/PDG@pMOF + L, G4: cApt/PDGs^s@pMOF + L, G5: Apt/PDGs^s@pMOF + L, G6: GEM + L, G7: Apt/PDGs^s@pMOF + D (dark). Significance is defined as ns, no significance, ⋆ P < 0.05, ⋆⋆ P < 0.01, ⋆⋆⋆P < 0.001.

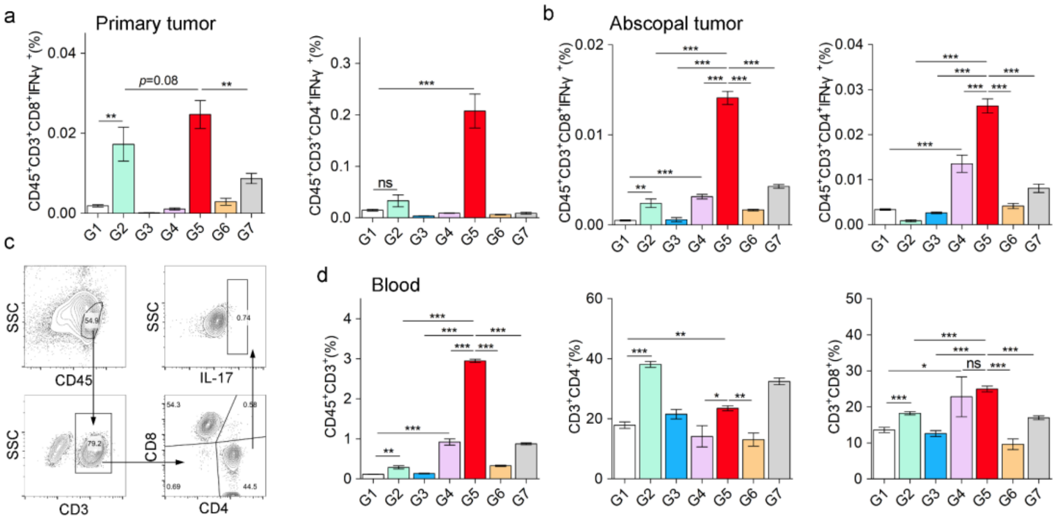

Figure S38. a. Active CTLs, Th1 cells levels in primary tumor lesions and abscopal tumor lesions (**b**), analyzed by flow cytometry (n=3). **c**. Gating strategy to determine frequencies of Th17 cells from bloods. **d**. $CD3^+$ T cells, $CD8^+$ T cells and $CD4^+$ T cells in blood of tumor-bearing mice, analyzed by flow cytometry (n=3). G1: Control + L (laser irradiation), G2: Apt/PDs^s@pMOF + L, G3: Apt/PDG@pMOF + L, G4: cApt/PDGs^s@pMOF + L, G5: Apt/PDGs^s@pMOF + L, G6: GEM + L, G7: Apt/PDGs^s@pMOF + D (dark). Significance is defined as ns, no significance, ⋆P < 0.05, ⋆⋆P < 0.01, ⋆⋆⋆P < 0.001.

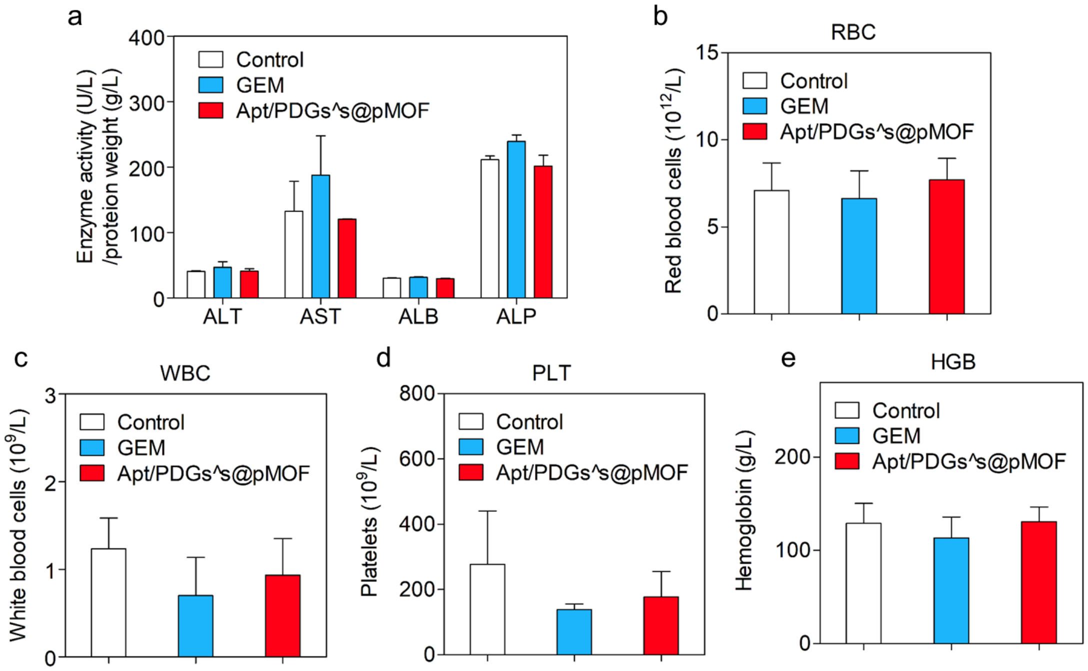

Figure S39. **a-e**, the concentrations/populations of alanine transaminase (ALT), aspartate transaminase (AST), serum albumin (ALB), alkaline phosphatase (ALP) (**a**), red blood cell (**b**), white blood cell (**c**), platelets (**d**), hemoglobin (**e**) in the blood of 12-week old female Balb/c mice after 12-day treatment with saline, gemcitabine (GEM), and Apt/PDGs^s@pMOF (administrated (*iv.*) with an equal dose of 200 μg/kg gemcitabine every four days for three times). There was no significant difference between Apt/PDGs^s@pMOF and saline treated mice. Data are presented as means ± SD (n=3).

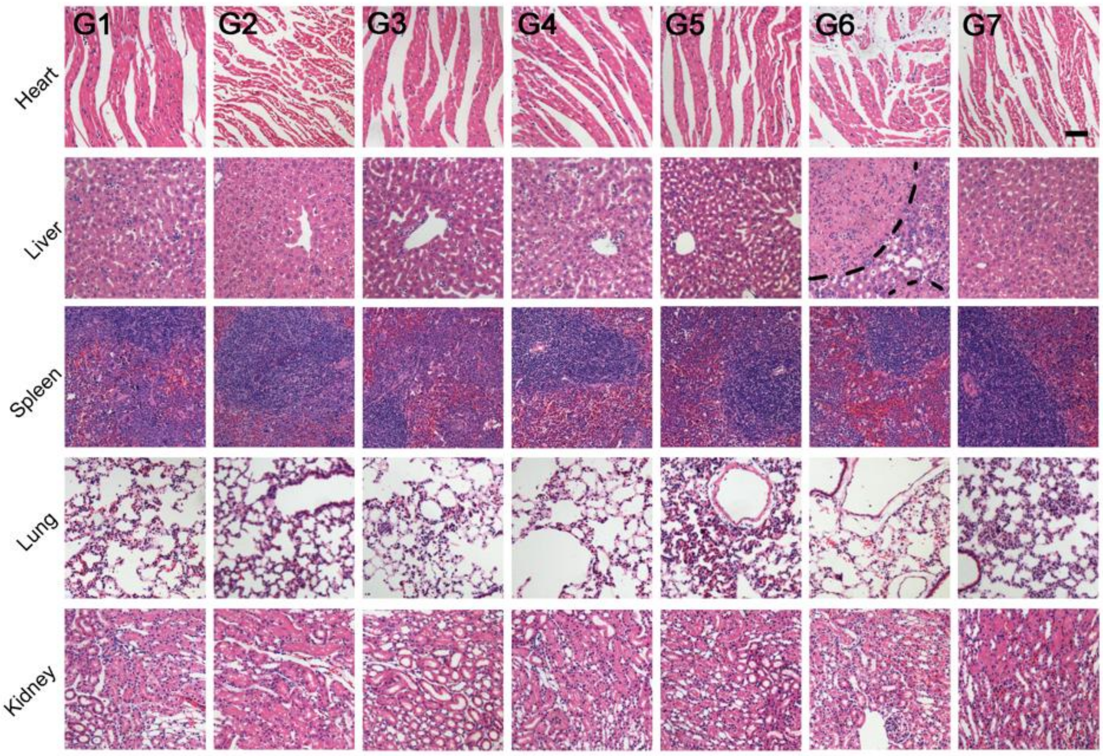

Figure S40. H&E staining images of major organ sections excised from mice treated with multiple formulations, and the diffuse hepatocyte necrosis was only found in liver in G6 groups, which was consistent with the literature reported that gemcitabine was hepatotoxic, as labeled in black line. Scale bar 50 μm. G1: Control + L (laser irradiation), G2: Apt/PDs^s@pMOF + L, G3: Apt/PDG@pMOF + L, G4: cApt/PDGs^s@pMOF + L, G5: Apt/PDGs^s@pMOF + L, G6: gemcitabine + L, G7: Apt/PDGs^s@pMOF + D (dark).

Target	Host	Source and catalog number	application
p-STAT3	Mouse	CST,4113S	WB, IF
PD-L1	Rabbit	Abcam, ab213480	IF
Calreticulin	Rabbit	Abcam, ab92516	WB, IF, Flow
HMGB1	Rabbit	Abcam, ab79823	WB, IF
CD34	Rabbit	Abcam, ab81289	IF
CD206	Rabbit	Abcam, ab64693	IF
CD16/32	Mouse	eBioscience, MA1-7633	IF
Rabbit IgG	Goat	Abcam, ab150077	IF, Flow
Mouse IgG	Goat	Abcam, ab150114	IF

CD45	Rat	eBioscience, 17-0451-82	Flow
CD8a	Rat	eBioscience, 12-0081-82	Flow
CD4	Rat	eBioscience, 45-0042-82	Flow
CD25	Rat	eBioscience, 53-0251-80	Flow
CD25	Rat	eBioscience, 25-0251-81	Flow
CD11c	Armenian hamster	eBioscience, 45-0114-80	Flow
CD80	Armenian hamster	eBioscience, 17-0801-82	Flow
CD86	Rat	eBioscience, 12-0862-82	Flow
CD3e	Armenian hamster	eBioscience, 25-0031-81	Flow
CD3	Rat	eBioscience, 11-0032-82	Flow
CD11b	Rat	eBioscience, 47-0112-80	Flow
Ly-6G/Ly-6C	Rat	eBioscience, 11-5931-81	Flow
Ly-6G	Rat	eBioscience, 12-9668-80	Flow
IFN-γ	Mouse	eBioscience, 48-7319-42	Flow
IL-17	Rat	eBioscience, 48-7177-80	Flow
Foxp3+	Rat	eBioscience, 56-4776-41	Flow
p-STAT3	Mouse	eBioscience, 48-9033-41	Flow

Table S3. Antibody information.
